# Adaptive Immune Response Signaling Is Suppressed in Ly6C^high^ Monocyte but Upregulated in Monocyte Subsets of *ApoE* ^-/-^ Mice — Functional Implication in Atherosclerosis

**DOI:** 10.3389/fimmu.2021.809208

**Published:** 2021-12-20

**Authors:** Pingping Yang, Qinghua Wu, Lizhe Sun, Pu Fang, Lu Liu, Yong Ji, Joon-Young Park, Xuebin Qin, Xiaofeng Yang, Hong Wang

**Affiliations:** ^1^ Department of Cardiovascular Medicine, The Second Affiliated Hospital of Nanchang University, Nanchang, China; ^2^ Center for Metabolic Disease Research, Department of Cardiovascular Science, Lewis Kats School of Medicine, Temple University, Philadelphia, PA, United States; ^3^ Department of Endocrinology, The Second Affiliated Hospital of Nanchang University, Nanchang, China; ^4^ Department of Cardiovascular Medicine, The First Affiliated Hospital of Xi’an Jiaotong University, Xi’an, China; ^5^ Key Laboratory of Cardiovascular Disease and Molecular Intervention, Nanjing Medical University, Nanjing, China; ^6^ Tulane National Primate Research Center, School of Medicine, Tulane University, Covington, LA, United States

**Keywords:** atherosclerosis, Ly6C MC, adaptive immune response, inflammatory, ApoE

## Abstract

**Rationale:**

Inflammatory monocyte (MC) subset differentiation is a major feature in tissue inflammatory and atherosclerosis. The underlying molecular mechanism remains unclear.

**Objective:**

This study aims to explore molecule targets and signaling which determinate immunological features in MC subsets.

**Methods and Results:**

Blood Ly6C^high^ and Ly6C^low^ MC subsets from control and *ApoE* ^-/-^ mice were isolated by flow cytometry sorting and subjected for bulk high-throughput RNA-sequencing. Intensive bioinformatic studies were performed by analyzing transcriptome through four pairs of comparisons: A) Ly6C^high^ vs Ly6C^low^ in control mice; B) Ly6C^high^ vs Ly6C^low^ in *ApoE^-/-^* mice; C) *ApoE^-/-^* Ly6C^high^ vs control Ly6C^high^ MC; D) *ApoE^-/-^* Ly6C^low^ vs control Ly6C^low^ MC. A total of 80 canonical pathways and 16 enriched pathways were recognized by top-down analysis using IPA and GSEA software, and further used for overlapping analysis. Immunological features and signaling were assessed on four selected functional groups, including MHCII, immune checkpoint, cytokine, and transcription factor (TF). Among the total 14578 significantly differentially expressed (SDE) genes identified though above four comparison, 1051 TF and 348 immunological genes were discovered. SDE immunological genes were matched with corresponding upstream SDE TF by IPA upstream analysis. Fourteen potential transcriptional axes were recognized to modulate immunological features in the Ly6C MC subset. Based on an intensive literature search, we found that the identified SDE immune checkpoint genes in Ly6C^high^ MC are associated with pro-inflammatory/atherogenic balance function. Immune checkpoint genes GITR, CTLA4, and CD96 were upregulated in Ly6C^low^ MC from all mice and presented anti-inflammatory/atherogenic features. Six cytokine genes, including Ccl2, Tnfsf14, Il1rn, Cxcl10, Ccl9, and Cxcl2, were upregulated in Ly6C^high^ MC from all mice and associated with pro-inflammatory/atherogenic feature. Cytokine receptor gene Il12rb2, Il1r1, Il27ra, Il5ra, Ngfr, Ccr7, and Cxcr5 were upregulated in Ly6C^low^ MC from all mice and presented anti-inflammatory/atherogenic features. MHCII genes (H2-Oa, H2-DMb2, H2-Ob, H2-Eb2, H2-Eb1, H2-Aa, and Cd74) were elevated in Ly6C^low^ MC from all mice. *ApoE* ^-/-^ augmented pro-atherogenic/inflammatory and antigen-presenting cells (APC) feature in both subsets due to elevated expression of cytokine genes (Cxcl11, Cntf, Il24, Xcl, Ccr5, Mpl, and Acvr2a) and MHCII gene (H2-Aa and H2-Ea-ps). Finally, we modeled immunological gene expression changes and functional implications in MC differentiation and adaptive immune response for MC subsets from control and *ApoE^-/-^* mice.

**Conclusions:**

Ly6C^high^ MC presented pro-inflammatory/atherogenic features and lower APC potential. Ly6C^low^ MC displayed anti-inflammatory/atherogenic features and higher APC potential. *ApoE* ^-/-^ confers upon both subsets with augmented pro-atherogenic/inflammatory function and APC potential.

## Introduction

Atherosclerosis is a chronic inflammatory disease of blood vessels and the essential pathological cause of cardiovascular disease, the leading cause of mortality worldwide (1, 2). Innate immune cell monocyte (MC) and macrophage (MΦ) is the major cellular components in the advanced atherosclerosis lesion, which is correlated with increased inflammatory MC differentiation (3–5). Emerging evidence supports the role of the adaptive immune system in atherosclerosis (6). However, the molecular mechanism underlying inflammatory MC differentiation, especially under hyperlipidemia (HL) conditions, and related adaptive immune response remains unknown.

MC circulates in the blood and migrates to inflammatory tissues, but their functions can be either detrimental or beneficial, determined by their subsets. Human CD14^++^CD16^+^ intermediate and CD14^+^CD40^+^ MC are considered as inflammatory MC subsets, similar to murine Ly6C^high^ and Ly6C^middle^ MC (7, 8). Human CD14^+^CD16^++^ nonclassical, CD14^++^CD16^-^ classical, and CD14^+^CD40^-^ MC are anti-inflammatory MC, similar as mouse Ly6C^low^ MC (7, 8).

In response to environmental stimulation, MC can be differentiated into different MC/MΦ subsets. Inflammatory MC/MΦ subsets are the major component in inflammatory tissue and advanced atherosclerosis. A recent study supported that the emerging roles of MC as antigen-presenting cells (APC) that enable adaptive immunity (9). Adaptive immunity is critical for disease progression and modulates T cell and APC functions, which was mediated by three concerted signals, including signal 1 antigen recognition, signal 2 immune checkpoint and signal 3 cytokine stimulation (10, 11). We recently proposed an additional novel signal 4, metabolism-associated danger signal recognition, which transmit metabolic risk factor response *via* metabolic sensor-mediated and pattern recognition leading to MC to APC differentiation and T cell activation (10, 11).

It is believed that inflammation triggers the differentiation of Ly6C^high^ MC into APC, mostly microbicidal MΦ or MC-derived dendritic cells (moDCs). Yet, little is known about the molecular mechanism determining Ly6C^high^ MC differentiation and their APC potential. Recently, we and others reported that TF NR4A1, CEBPα, CEBPβ, and PU.1 are involved in the process of Ly6C^high^ MC differentiation to Ly6C^low^ MC (3, 12, 13). The activation of STAT3 was described in MC-to-MΦ differentiation and inflammation (14). It was shown that major histocompatibility complex II (MHCII) was elevated in Ly6C^high^ MC after infiltrating into the skin or skin-draining lymph nodes (15). Through transcriptome analysis in sorted mouse Ly6C MC subset, we reported that the Ly6C^high^ MC displayed enriched inflammatory pathways and favored to be differentiated into MΦ and osteoclast (13). However, molecular mechanism underlying MC subset differentiation, specially under HL condition, remains to be elucidated.

MC/MΦ account for about 65% of CD45^+^ immune cells in advanced atherosclerotic aorta in human (16). MC/MΦ population is up to about 48.1% of CD45^+^ cells and the major immune cell populations in lesion of hyperlipidemic *ApoE* ^-/-^ and *Ldlr* ^-/-^ mice, and their combination with hyperhomocysteinemia (HHcy) cystathionine b-synthase gene-deficient (*Cbs-/-*) (4, 16–18). The myeloid response is accompanied by the infiltration of adaptive immune cells (18). In atherosclerosis-prone *ApoE* ^-/-^CD11c^-^YFP^+^ mice, APC and CD4^+^ T helper cell interactions were increased in the plaque that resulted in pro-inflammatory cytokine IFN-γ and TNF-α secretion (19). Through transcriptome analysis, we recently revealed that Ly6C^low^ MC is enriched with genes manifesting T cell activation signaling in wild type and *Cbs-/-* mice (13). Although the potential role of APC and adaptive immune cells interaction in atherosclerosis is known, the specific molecular mechanism in regulating innate/adaptive immunity in disease conditions remains unclear.

This study aims to identify molecular mechanisms underlying MC subset differentiation, with a focus on HL-induced MC differentiation and innate/adaptive interplay, by analyzing MC subset transcriptome using intensive bioinformatic studies.

## Research Design and Methods

**Figure 1:**
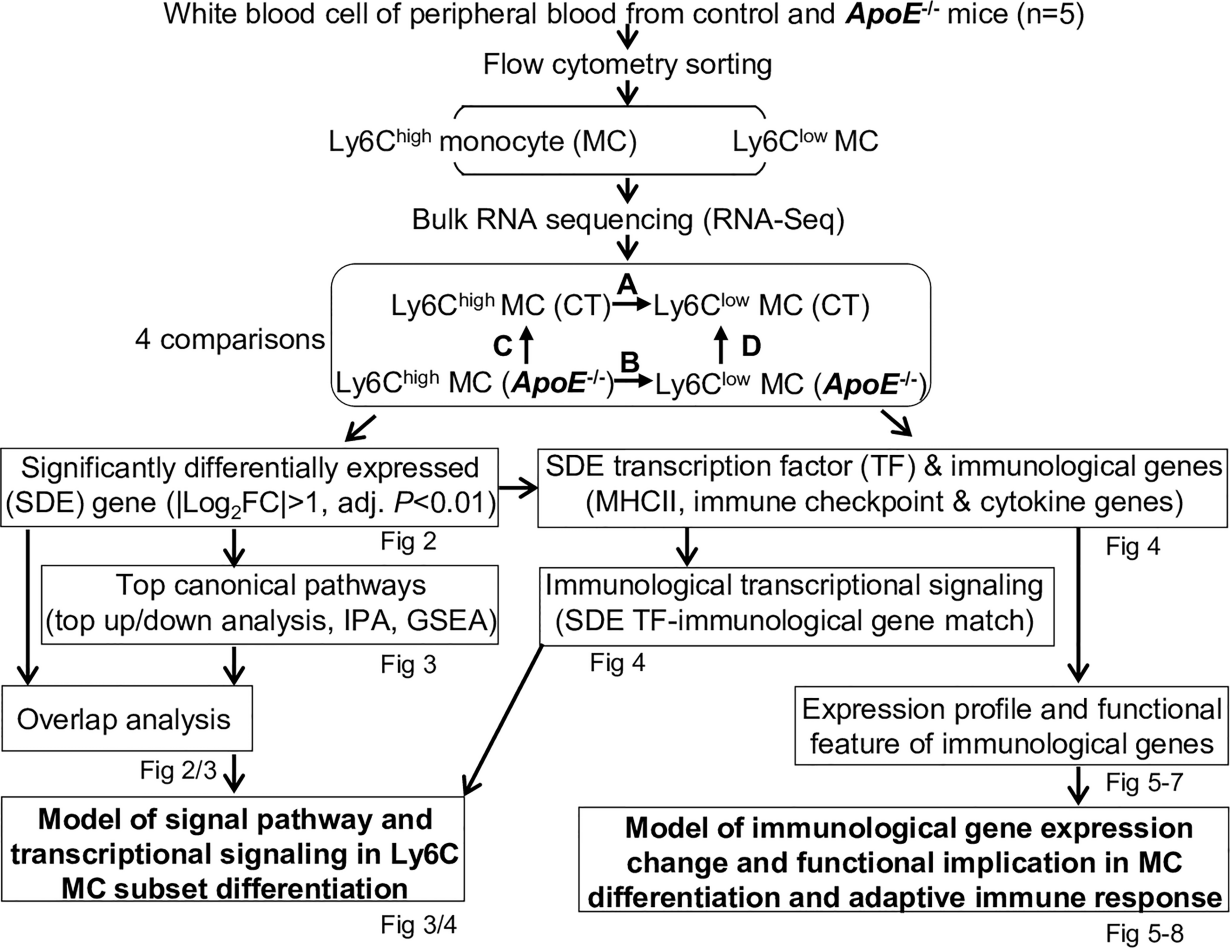
Overall strategy of the identification of molecular signaling in Ly6C MC subset differentiation and adaptive immune response in control and *ApoE* ^-/-^ mice. RNA-Seq were performed in Ly6C^high^ (CD11b^+^Ly6G^−^Ly6C^high^) and Ly6C^low^ (CD11b^+^Ly6G^−^Ly6C^low^) MC isolated by flow cytometry sorting from peripheral blood of C57/BL6 control and *ApoE* ^-/-^ mice. Transcriptome data were analyzed by performing four pairs of comparisons: **(A)** Ly6C^high^ vs Ly6C^low^ (CT), **(B)** Ly6C^high^ vs Ly6C^low^ (*ApoE^-/-^*), **(C)** *ApoE^-/-^* vs CT (Ly6C^high^), **(D)** *ApoE^-/-^* vs CT (Ly6C^low^). SDE genes were identified by using the criteria of |Log_2_FC| more than 1 (2-FC) and adjusted P value less than 0.01. Top canonical pathways were recognized by top-down analysis using IPA with |Z-score|>2, *P* value<0.05. Overlapped analysis were performed for canonical pathways between groups. SDE TF and three sets of SDE immunological genes (MHCII, immune checkpoint and cytokine genes) were identified. Immunological SDE genes were matched with corresponding upstream SDE TF by IPA upstream analysis. Models of signal pathway and transcriptional signaling of Ly6C MC subset differentiation were developed. Expression profile and functional feature of immunological SDE genes were characterized. Model of immunological gene expression change and functional implication in MC differentiation and adaptive immune response in MC subsets of both mice were developed. CT, control, ApoE, Apolipoprotein E; FC, fold change; RNA-seq, RNA-sequencing; MC, monocyte; SDE, significant differentially expressed; IPA, Ingenuity Pathway Analysis; GSEA, gene set enrichment analysis; TF, transcription factor.

We summarized the overall study approaches and strategies in **Figure 1**.

### *ApoE^-/-^* Mice and Plasma Lipid Determination

— *ApoE^-/-^* mice were fed a normal chow diet and switched to a high-fat diet [21% fat (w/w), 0.15% cholesterol (w/w); Dyets Inc., Bethlehem, PA] at age 8 weeks and maintained on a high-fat diet for 12 weeks. The control (CT) C57BL/6 mice were fed a normal chow diet throughout. Animals were sacrificed at 20-22 weeks of age for blood collection after euthanization. Mouse blood was collected into 1 mM ethylenediaminetetraacetic acid (EDTA)-coated tubes for MC and plasma preparation. The plasma was separated (3,000g for 20 min). Plasma total cholesterol and triglyceride (TG) were analyzed as we previously described (20). All experiments were conducted in accordance with the National Institutes of Health *Guidelines for the Care and Use of Laboratory Animals* and with approval from Temple University School of Medicine Institutional Animal Care and Use Committee.

### Flow Cytometry and Cell Sorting

MC from mouse peripheral blood were collected and sorted by flow cytometry for Ly6C^high^ (CD11b^+^Ly6G^−^Ly6C^high^) and Ly6C^low^ (CD11b^+^Ly6G^−^Ly6C^low^) MC from CT and *ApoE* ^-/-^ cells isolation as described (13) and detailed in a supplementary document. The difference of Ly6C^high^ and Ly6C^low^ MC between two groups of CT and *ApoE* ^-/-^ after sorting was shown in the **Supplementary Figure 1**.

### RNA Sequencing and Data Processing

RNA was extracted. Pooled samples (5-6 in each) were run for sequencing analysis in duplication on NextSeq 500 (CT) and Illumina Hiseq 4000 sequencer (*ApoE* ^-/-^). Overall, we obtained around 40 million reads per sample. The raw RNA-seq data were analyzed using R and RStudio. The raw reads were mapped to the mouse reference transcriptome using Kallisto as described (13) and detailed in a supplementary document.

### Bioinformatic Analysis and Model Establishment

Intensive bioinformatic analysis was performed as described (13) and detailed in a supplementary document. Briefly, principle components analysis (PCA) was performed to examine the variance of RNA-seq data. Significantly differentially expressed (SDE) genes were identified by using the Bioconductor suite of Limma packages in RStudio software with the criteria of |fold change (FC)|>2 and adjusted *P*-value<0.01. Heatmap was generated in RStudio using the “pheatmap” package to present the expression levels of SDE genes. Ingenuity Pathway Analysis (IPA) version 7.1 was used to identify functional pathways and matched SDE TF with their corresponding SDE immunological genes. Gene set enrichment analysis (GSEA) was used for pathway enrichment study. Venn diagrams were displayed to present the overlaps of SDE genes and pathways between comparisons. Finally, we developed models of hypothetic signal pathway and transcriptional signaling of Ly6C MC subset differentiation and immunological functional implication.

## Results

### RNA-Seq Analysis and SDE Gene Identification From Blood Ly6C^high^ and Ly6C^low^ MC of CT and *ApoE* ^-/-^ Mice

**Figure 2:**
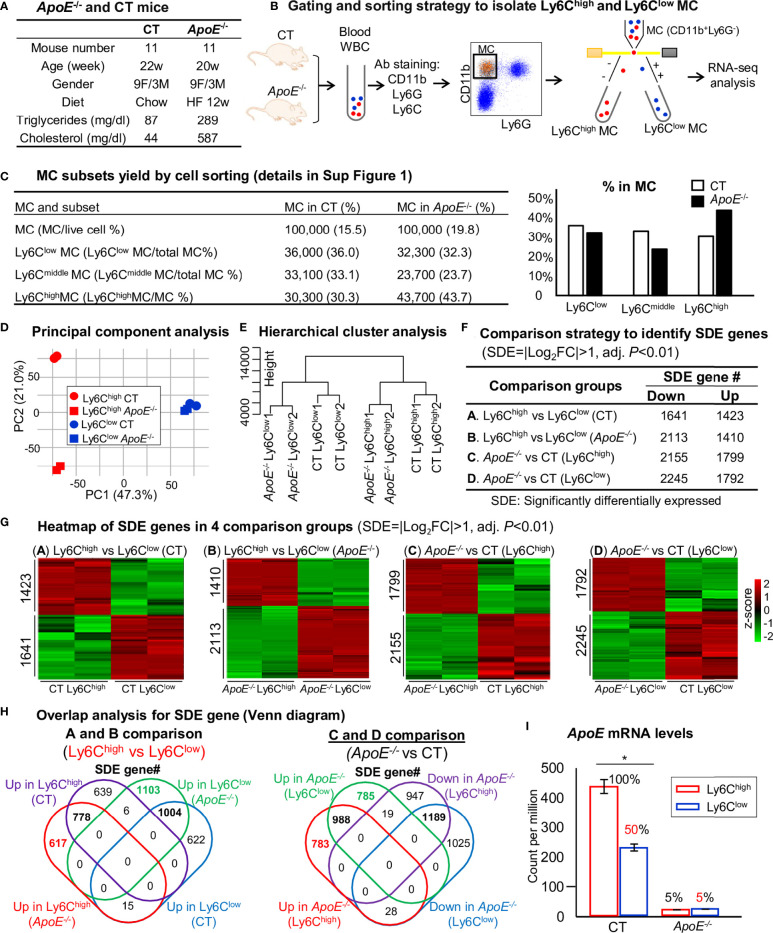
RNA-Seq analysis and SDE gene identification from blood Ly6C^high^ and Ly6C^low^ MC of control and *ApoE* ^-/-^ mice. **(A)** *ApoE* ^-/-^ and CT mice. Eleven mice at the age of 20 weeks were used in each group. C57/BL6 mice on rodent chaw were used as CT. *ApoE* ^-/-^ mice were fed a high-fat diet for 8 weeks. **(B)** Gating and sorting strategy to isolate Ly6C^high^ and Ly6C^low^ MC. Mouse white blood cell were prepared from peripheral blood, pooled, stained with antibody against CD11b, Ly6G and Ly6C and subjected for flow cytometry cell sorting. CD11b^+^Ly6G^-^ cells were characterized as MC. MC subsets (CD11b^+^Ly6G^-^Ly6C^high^, and CD11b^+^Ly6G^-^Ly6C^low^) were sorted and used for bulk RNA-seq analysis. **(C)** MC subsets yield by cell sorting. MC (100,000 cells) were sorted by flow cytometry with 43.7% Ly6C^high^, 23.7% Ly6C^middle^ and 32.3% Ly6C^low^ MC from *ApoE* ^-/-^ mice, and 30.3% Ly6C^high^, 33.1% Ly6C^middle^ and 36% Ly6C^low^ MC in CT mice. The detail of flow cytometry sorting cell subset was presented in **Supplementary Figure 1**. **(D)** Principal component analysis. PCA analysis incorporated 8 samples from 4 groups of MC subsets {Ly6C^high^ (CT), Ly6C^low^ (CT), Ly6C^high^ (*ApoE^-/-^*) and Ly6C^low^ (*ApoE^-/-^*)}. **(E)** Hierarchical cluster analysis. The similarity of gene expression between different samples is represented by the vertical distances on each branch of the dendrogram. Biological replicates show the highest degree of correlation within samples, represented by short vertical distances. **(F)** Comparison strategy to identified SDE genes. Four group comparisons **(A–D)** were performed. Down-regulated and up-regulated SDE genes were identified using the criteria of |Log_2_FC| more than 1 (2-FC) and adjusted P value less than 0.01. **(G)** Heatmap of SDE gene in 4 comparison groups. Heatmap shows the expression levels of the SDE gene in Ly6C MC. The color density indicates the average expression of a given gene normalized by z-score. **(H)** Overlap analysis for SDE gene. Venn diagram summarized the total SDE genes from four pairs of comparisons. Numbers depict the amount of SDE genes. **(I)** *ApoE* mRNA levels. MC, monocyte; CT, wild type; *ApoE*, Apolipoprotein E; FACS, fluorescent-activated cell sorting; PCA, principal component analysis; PC, principal component; SDE, significantly differentially expressed; FC, fold change. *P < 0.05.

Eleven mice at age of 20 weeks were used. Severe HL were developed in *ApoE* ^-/-^ mice fed an HF diet for 12 weeks (plasma TG 289 mg/dl and cholesterol 587 mg/dl) (**Figure 2A**). In contrast, C57BL/6 CT animals had plasma TG of 87 mg/dl and cholesterol of 44 mg/dl. RNA-seq was performed in Ly6C^high^ (CD11b^+^Ly6G^−^Ly6C^high^) and Ly6C^low^ (CD11b^+^Ly6G^−^Ly6C^low^) MC isolated by flow cytometry sorting from mouse peripheral blood (**Figure 2B**). MC (100,000 cells) were sorted by flow cytometry with 43.7% Ly6C^high^, 23.7% Ly6C^middle^ and 32.3% Ly6C^low^ MC from *ApoE* ^-/-^ mice, and 30.3% Ly6C^high^, 33.1% Ly6C^middle^ and 36% Ly6C^low^ MC in CT mice (**Figure 2C**, details in **Supplementary Figure 1**)*. ApoE* ^-/-^ elevated Ly6C^high^ and reduced Ly6C^middle/low^ subsets. Principal component (PC) analysis (PCA) incorporated 8 samples from 4 groups of MC subsets [Ly6C^high^ (CT), Ly6C^low^ (CT), Ly6C^high^ (*ApoE* ^-/^ *^-^*) and Ly6C^low^ (*ApoE* ^-/-^)]. PC1, PC2 and PC3 variance is 47.3%, 21.0% and 13.6% (**Figure 2D**). The PC1 axis separated Ly6C^high^ and Ly6C^low^ MC in both mice, and explains 47.3% of the variance. Although the PC2 axis, which explains 21.0% of the variance, separated the Ly6C^high^ MC from the *ApoE* ^-/-^ and CT groups, but not the Ly6C^low^ MC. However, the hierarchical clustering well separated the MC subsets in both groups (**Figure 2E**). We identified 1423-upregulated/1641-downregulated SDE genes, and 1410-upregulated/2113-downregulated SDE genes in Ly6C^high^ MC from CT and *ApoE* ^-/-^ mice by A/B comparison. HL in *ApoE* ^-/-^ mice with 12-weeks HF diet-induced 1799-upregulated/2155-downregulated SDE gene in Ly6C^high^ MC and induced 1792- upregulated/2245-downregulated SDE gene in Ly6C^low^ MC (**Figures 2F, G** **)**.

By overlap analysis, we identified 778 upregulated and 1004 downregulated SDE gene in Ly6C^high^ MC in CT and *ApoE* ^-/-^ mice (**Figure 2H**). HL in *ApoE* ^-/-^ mice induced 988 upregulated and 1189 downregulated SDE gene in both Ly6C^high^ and Ly6C^low^ MC. Interestingly, *ApoE* mRNA levels were significantly reduced to 50% in Ly6C^low^ MC compared with that in Ly6C^high^ MC in CT mice, and depleted to about 5% in *ApoE* ^-/-^ mice (**Figure 2I**).

### Ly6C^low^ MC Engages More Adaptive Immune Function

**Figure 3:**
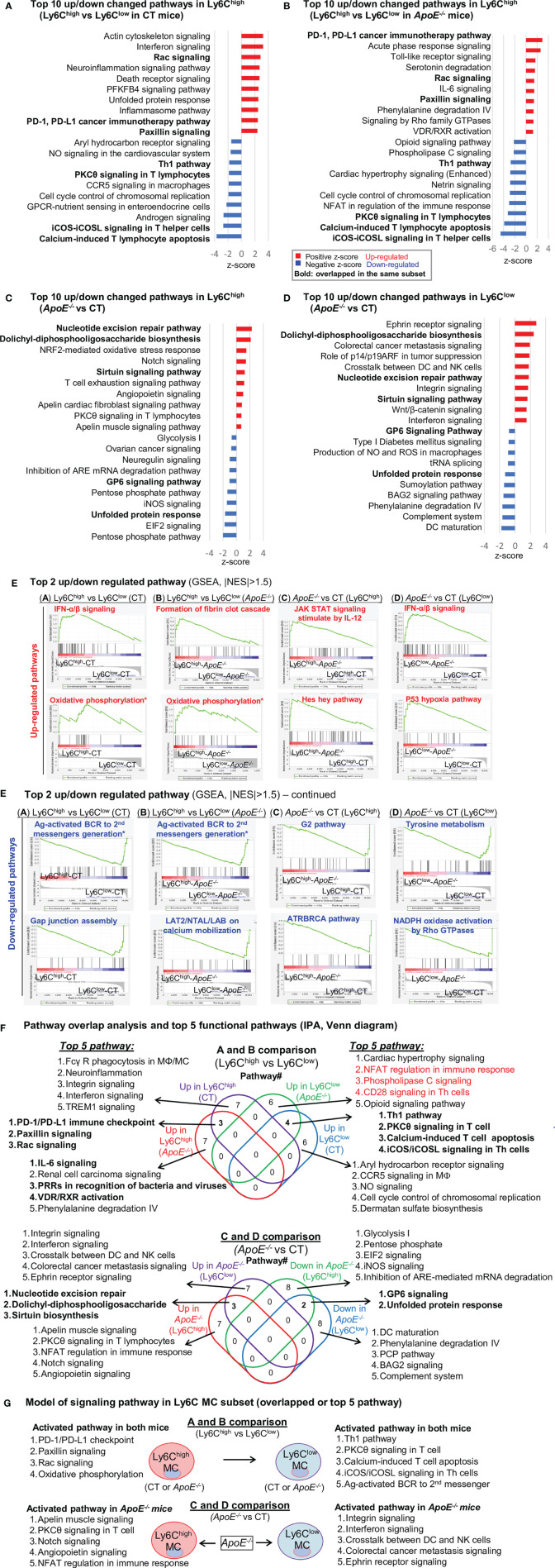
Canonical pathway analysis for SDE genes in four comparison groups (Top 10 up/down changed pathways), **(A)**. CT Ly6C^high^ vs CT Ly6C^low^; **(B)**. *ApoE^-/-^* Ly6C^high^ vs *ApoE^-/-^* Ly6C^low^; **(C)**. Ly6C^high^ (*ApoE^-/-^* vs CT); **(D)**. Ly6C^low^ (*ApoE^-/-^* vs CT). Top up/down changed canonical pathways were identified by using IPA software with the criteria of adjusted P value<0.05. Blue bar indicates a negative z-score and down-regulated pathways. Red bar indicates a positive z-score and up-regulated pathways. Bold letters indicate overlapped pathway in the same subset. **(E)** Top 2 up/down regulated pathway. The most enriched significant pathways from GSEA study with threshold of |NES|>1.5 are marked (red=up-regulated, blue=down-regulated). The green curve corresponds to the enriched score. The y-axis indicates the enriched score. The x-axis displaces genes (vertical black lines) represented in their pathway gene set. Rainbow bands represent the corresponding enriched score of the genes (red for positive and blue for negative correlation). The top 20 upregulated or downregulated GSEA pathways were shown in **Supplementary Table 1**. **(F)** Pathway overlap analysis and top 5 functional pathways. Venn diagram summarized the overlap of top 10 pathway presented in **(A–D)** and listed the top 5 pathways in four pairs of comparisons. **(G)** Model of signaling pathway in Ly6C MC subset (overlapped or top 5 pathway). Four activated pathways in Ly6C^high^ MC and 5 activated pathway in Ly6C^low^ MC were overlapped in A and B comparison. *ApoE* ^-/-^ induced top 5 activated pathway in both Ly6C^high^ MC and Ly6C^low^ MC. MC, monocyte; MΦ, macrophage; TREM1, The triggering receptor expressed on myeloid cells 1; GPCRs, G-protein-coupled receptors; PFKFB4, 6-phosphofructo-2-kinase/fructose-2,6-biphosphatase 4; SLE, Systemic Lupus Erythematosus, Th1, T helper 1; PKCθ, Protein Kinase C Theta; IL-7, Interleukin 7; NFAT, Nuclear factor of activated T-cells; CHK, Csk-homologous kinase; nNOS, neuronal nitric oxide synthase; PXR, pregnane X receptor; CAR, constitutive androstane receptor. NO, Nitric Oxide; ROS, Reactive Oxygen Species; PRRs, Pattern Recognition Receptors.

We recognized top 10 up/down canonical pathways that were significantly enriched by top-down analysis using SDE genes identified from four comparison groups by using IPA software (**Figures 3A–D**). The top 2 up/down enrichment pathways from the GSEA study in each group are presented in **Figure 3E**. The top 20 upregulated or downregulated GSEA pathways were shown in **Supplementary Table 1**. The GSEA study showed that OXPHOS pathway activation and antigen-activated B cell receptor to second messengers’ generation pathway suppression were overlapped in Ly6C^high^ MC from both CT and *ApoE* ^-/-^ mice. In addition, interferon (IFN) α/β signaling was upregulated and Gap junction assembly signaling was downregulated in Ly6C^high^ MC from CT mice. Formation of fibrin clot cascade was upregulated and LAT2/NTAL/LAB on calcium mobilization signaling were downregulated in *ApoE* ^-/-^ Ly6C^high^ MC. HL in *ApoE* ^-/-^ mice induced IL-12-stimulated JAK-STAT signaling and Hes hey pathway activation and G2 and ATRBRCA pathway suppression in Ly6C^high^ MC. In Ly6C^low^ MC, *ApoE* ^-/-^ induced IFN-α/β signaling and P53 hypoxia pathway activation, and suppressed Tyrosine metabolism and Rho GTPases-related NADPH oxidase activation.

Through IPA pathway overlap analysis (**Figure 3F**), among the top 10 upregulated pathways from comparison A and B, 3 pathways (PD-1/PD-L1 checkpoint, paxillin, and Rac signaling) were overlapped in Ly6C^high^ MC from both CT and *ApoE* ^-/-^ mice, and 7 were only activated in either mouse. *ApoE* ^-/-^ upregulated IL-6, Renal cell carcinoma, PRRs in recognition of bacteria and viruses, VDR/RXR activation, Phenylalanine degradation IV pathways in Ly6C^high^ MC.

Among the top 10 downregulated pathways from comparison A and B, 4 pathways (Th1 pathway, PKCθ in T cell, calcium-induced T cell apoptosis and iCOS/iCOSL in Th cells) were overlapped in Ly6C^high^ MC from both CT and *ApoE* ^-/-^ mice, and 7 were only suppressed in either mouse. *ApoE* ^-/-^ downregulated NFAT regulation in immune response, Phospholipase C and CD28 (Th cells) pathways in Ly6C^low^ MC.

From comparison C and D (**Figure 3F**), we identified *ApoE* ^-/-^ alter pathways in both subsets. *ApoE* ^-/-^ activated 3 pathways (nucleotide excision repair, dolichyl-diphosphooligosaccharide, sirtuin biosynthesis) and suppressed 2 pathways (GP6 and unfolded protein response) in both Ly6C^high^ and Ly6C^low^ MC. In addition, *ApoE* ^-/-^ specifically activated 5 pathways (Apelin, PKCθ, NFAT regulation, notch and angiopoietin), and suppressed 5 pathways (glycolysis I, pentose phosphate, EIF2 and iNOS) only in Ly6C^high^ MC. Whereas, *ApoE* ^-/-^ specifically activated 5 pathways (integrin, interferon, crosstalk between DC and NK cells, colorectal cancer metastasis, ephrin receptor) and suppressed 5 pathways (phenylalanine degradation IV, PCP, BAG2 and complement system) only in Ly6C^low^ MC. Finally, we established a model to describe specific pathways in the Ly6C MC subset based on IPA and GSEA pathway analysis (**Figure 3G**).

### Fourteen Potential Transcriptional Axes Modulate Immunological Feature in Ly6C MC Subset From CT and *ApoE* ^-/-^ Mice

**Figure 4:**
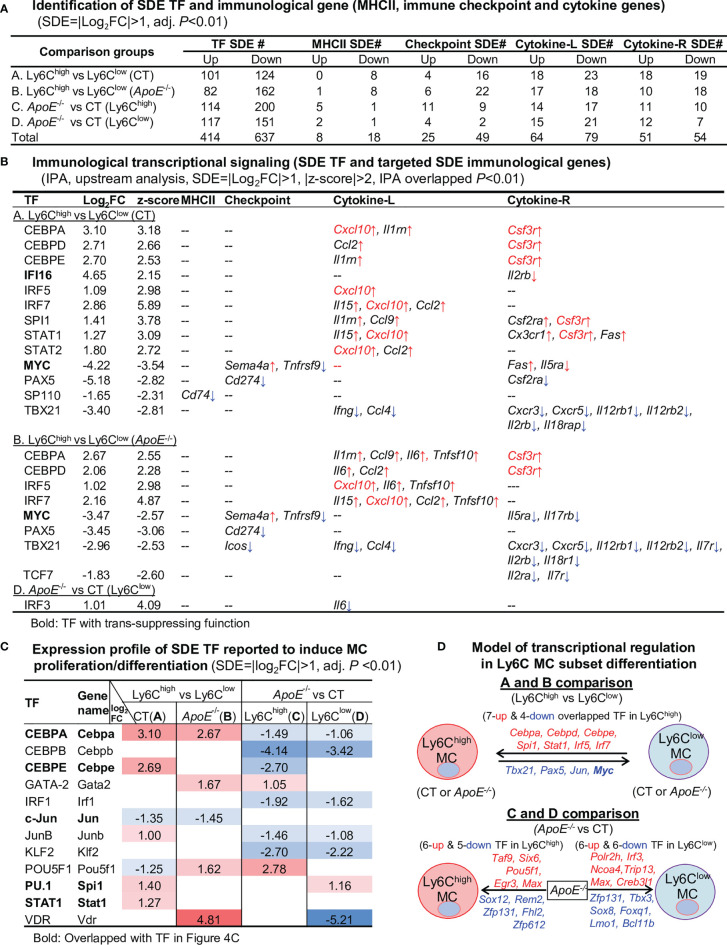
Identification of SDE TF, immunological gene and transcriptional regulatory models. **(A)** Identification of SDE TF and immunological genes. SDE TF (1150) and MHCII (28), immune checkpoint (82), cytokine ligand (135) and receptor (89) were identified using the criteria of |Log_2_FC|>1 (2-FC) and adjusted P<0.01. **(B)** Immunological transcriptional signaling. SDE immunological genes were matched with SDE TF by IPA upstream analysis. Transcriptional regulatory relationship between SDE TF and SDE immunological genes was justified by p-value<0.01 and |z-score|>2. The detailed list of SDE TF matching with the corresponding SDE gene is presented in **Supplementary Table 2**. ↑, upregulated; ↓, downregulated **(C)** Expression profile of SDE TF reported to induce MC proliferation/differentiation. Twelve SDE TF involved in MC generation are differentially expressed in four comparison groups in these subsets. Numbers with red-colored background indicate fold change>2 (log_2_FC>1). Numbers with blue-colored background indicate fold change<0.5 (log_2_FC<-1). The completed list of TF reported to induce MC proliferation/differentiation is in **Supplementary Table 3**. **(D)** Model of transcriptional regulation in Ly6C MC subset differentiation. Model describes potential transcriptional regulatory machinery. In A and B comparison, we identified 7-upregulated and 5-downregulated overlapped TF regulating Ly6C MC differentiation. In comparison C, 11 SDE TF (6 up and 5 down) are identified in *ApoE^-/-^* Ly6C^high^ MC. While, in comparison D, 12 SDE TF (6 up and 6 down) are identified in *ApoE^-/-^* Ly6C^low^ MC. Red letter highlighted the representative up-regulated gene. Blue letter highlighted down-regulated genes.

In the efforts to discover transcriptional signal determining immunological feature in Ly6C MC subset, we identified 414-upregulated/637-downregulated TF, 8-upregulated/18-downregulated MHC II, 25-upregulated/49-downregulated checkpoint, 64-upregulated/79-downregulated cytokine ligand, and 51-upregulated/54-downregulated cytokine receptor SDE genes from the 4 comparisons (details in **Figure 4A**).

By matching the immunological SDE genes with their corresponding upstream SDE TF by using IPA upstream analysis, we identified 12 trans-activating TF (activating transcription factor), one trans-suppressing TF (transcription repressors) IFI16, and one dual-functional TF MYC, which potentially regulate the identified SDE immunological genes transcription (**Figure 4B**). In A comparison, there are 8 upregulated SDE trans-activating TF (CEBPA, CEBPD, CEBPE, IRF5/7, PU.1 and STAT1/2) corresponding to 9 upregulated cytokine-L/R, and 3 downregulated SDE trans-activating TF (PAX5, SP110 and TBX21) corresponding to 10 downregulated immunological gene in CT Ly6C^high^ MC. We also found one upregulated transcription repressors IFI16, which was associated with the downregulation of Il2rb. The downregulated dual functional TF MYC (21, 22) was associated with Tnfrsf9/Il5ra downregulation and Sema4a/Fas upregulation. In B comparison, 4 upregulated SDE trans-activating TF (CEBPA, CEBPD and IRF5/7) was associated with 8 upregulated cytokine/receptors in *ApoE* ^-/-^ Ly6C^high^ MC. Three SDE trans-activating TF (PAX5, TBX21 and TCF21) were downregulated and corresponded to 12 downregulated immunological gene in *ApoE* ^-/-^ Ly6C^high^ MC. TF MYC downregulation was associated with Tnfrsf9/Il5ra/Il17rb downregulation and Sema4a upregulation. In D comparison, we identified upregulated SDE trans-activating TF IRF3 corresponding to upregulated cytokine-L IL6 in *ApoE* ^-/-^ Ly6C^high^ MC. The detailed list of SDE TF matching with the corresponding SDE gene is presented in **Supplementary Table 2**.

Among the matched SDE TF identified in **Figure 4B**, 12 were validated with conformed function in regulating MC proliferation/differentiation (**Figure 4C**, details in **Supplementary Table 3**). Apparently, these validated SDE TF were mostly upregulated in Ly6C^high^ MC, and largely downregulated by *ApoE* ^-/-^ in both Ly6C^high^ and Ly6C^low^ MC. We established models to describe potential transcriptional regulatory machinery in Ly6C MC subset differentiation in **Figure 4D**.

### Immune Checkpoint Genes Present Balanced Pro-Inflammatory/Atherogenic Features in Ly6C^high^ MC, and Anti-Inflammatory/Atherogenic Features Ly6C^low^ MC

**Figure 5:**
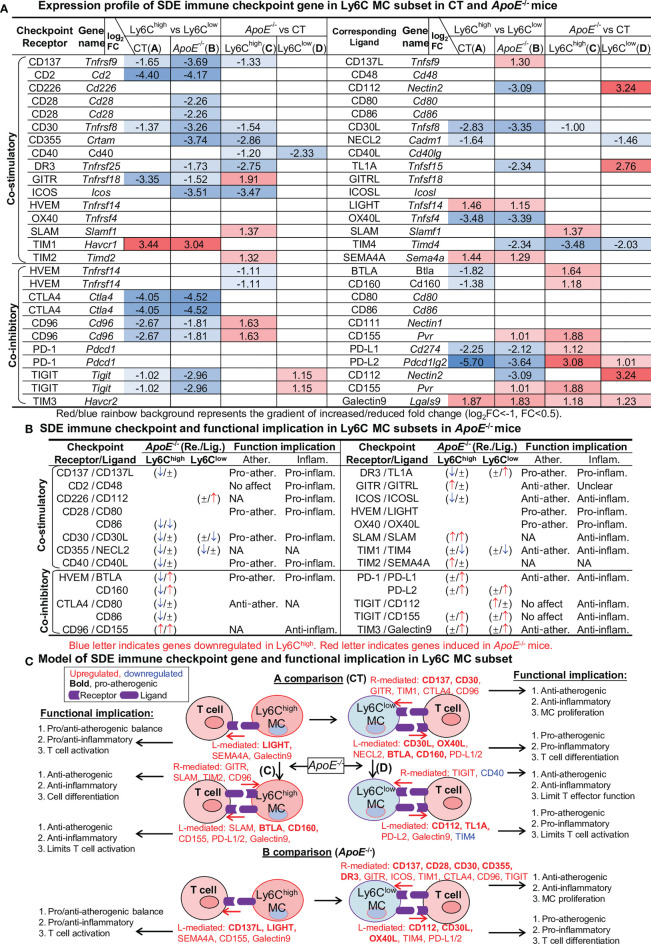
Identification of SDE immune checkpoint gene and function implication in Ly6C MC subset in CT and *ApoE* ^-/-^ mice. **(A)** Expression profile of SDE immune checkpoint gene. Sixteen pairs of SDE co-stimulatory and 11 pairs of SDE co-inhibitory molecules are identified in four comparison groups. **(B)** SDE immune checkpoint gene functional implication in Ly6C MC subsets in *ApoE* ^-/-^ mice. ↑ refers to induce expression by *ApoE* ^-/-^. ↓ refers to reduce expression by *ApoE* ^-/-^, ± refers to no changes in *ApoE* ^-/-^. **(C)** Model of SDE immune checkpoint gene and functional implication. Red letter highlighted the representative up-regulated gene. Blue letter highlighted down-regulated genes. Bold letter emphasized pro-atherogenic function. NA, Not applicable.

To examine the differential role of Ly6C MC subsets in regulating adaptive immunity, we analyzed the expression pattern of immune checkpoint molecules. As depicted in **Figure 5A**, 27 out of 49 checkpoint pairs displayed differential expression in Ly6C MC subsets. In general, Ly6C^high^ MC expressed relatively low levels of both co-stimulatory and co-inhibitory immune checkpoint receptors compared to Ly6C^low^ in both mice, except for TIM1. *ApoE* ^-/-^ upregulated immune checkpoint receptor GITR, SLAM, TIM2 and CD96 in Ly6C^high^ and TIGIT in Ly6C^low^ MC subsets. We identified a trend of downregulation of some immune checkpoint ligands genes in Ly6C^high^ compared to Ly6C^low^ MC in both mice. *ApoE* ^-/-^ upregulate immune checkpoint ligands SLAM, BTLA, CD160, CD155, PD-L1, PD-L2, CD155 and Galectin9 in Ly6C^high^ and CD112, TL1A, PD-L2 and Galectin9 in Ly6C^low^ MC subsets.

Through an intensive literature search, we established the functional implication relevant to atherosclerosis and inflammation of these differentially expressed checkpoint genes in *ApoE* ^-/-^ Ly6C^high^ and Ly6C^low^ MC **(** **Figure 5B**). Finally, we modeled this functional implication in **Figure 5C**.

### Cytokine Expression Profile Endued Ly6C^high^ MC With Pro-Inflammatory/Atherogenic Function and Ly6C^low^ MC With Anti-Atherogenic Function. *ApoE* ^-/-^ Confer Additional Pro-Atherogenic/Inflammatory Function

**Figure 6:**
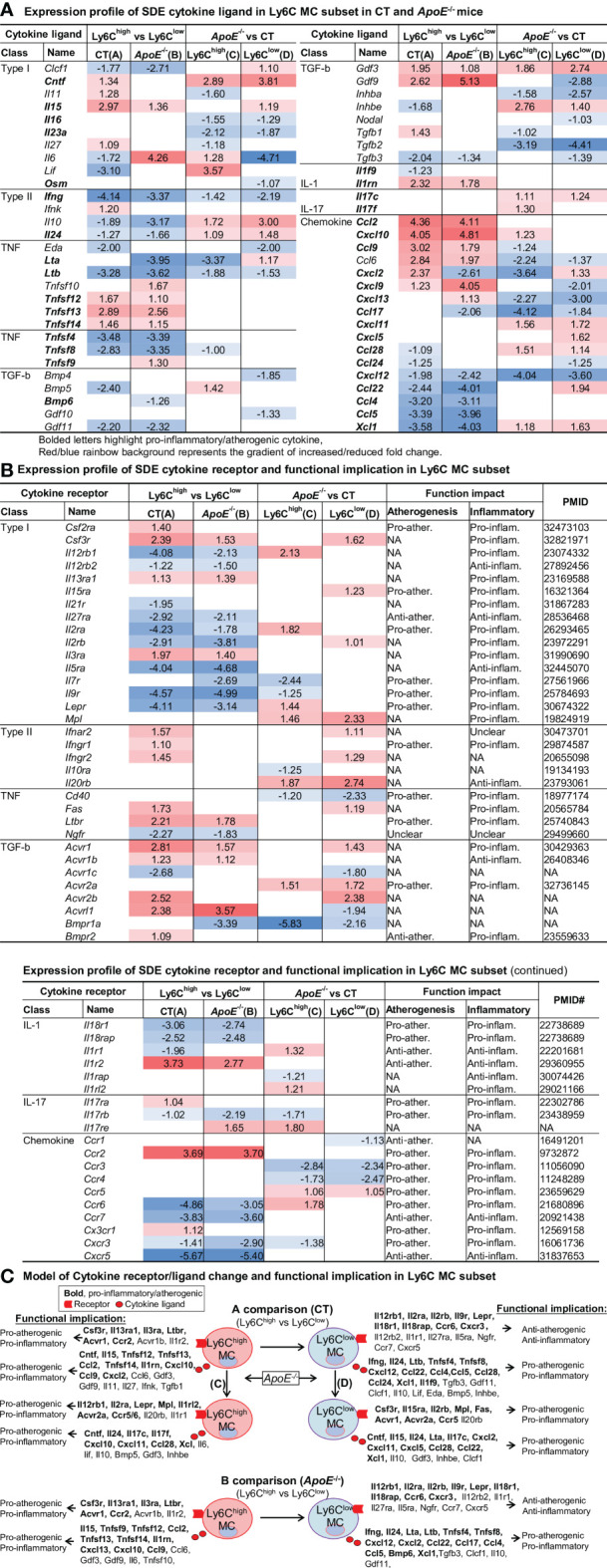
Identification of SDE cytokine gene and function implication in Ly6C MC subset in CT and *ApoE* ^-/-^ mice. **(A)** Expression profile of SDE cytokine ligand. Fifty-eight SDE cytokine ligand are identified in four comparison groups. **(B)** Expression profile of SDE cytokine receptor and function implication. Fifty-two SDE cytokine receptor are identified in four comparison groups. There function implication are summarized based on literature search (PMID cited). Red/blue rainbow background represents the gradient of increased/reduced fold change (log_2_FC>1 and log_2_FC<-1, respectively). **(C)** Model of cytokine receptor/ligand change and functional implication. Functional implication is conclude based on cytokine ligand and receptor change. Bold letter emphasized pro-inflammatory and pro-atherogenic function. NA, Not applicable.

To test the differential role of Ly6C MC subsets in regulating inflammatory response, we further examined differential expression of cytokine ligand and receptor in MC subsets. The identified 58 SDE cytokine ligand and expression profile are presented in **Figure 6A**. The expression profile of 52 SDE cytokine receptors and their functional implication established through literature search are exhibited in **Figure 6B**. We modeled the inflammatory/atherogenic functional implication of cytokine in Ly6C MC subsets in **Figure 6C**.

We identified that Ly6C^high^ MC expressed elevated pro-inflammatory/atherogenic cytokines (Ccl2, Tnfsf14, Il1rn, Cxcl10, Ccl9, Cxcl2), and receptors (Csf3r, Il13ra1, Il3ra, Ltbr, Acvr1 and Ccr2). Whereas Ly6C^low^ MC expressed high levels of anti-atherogenic function cytokine receptor (Il12rb2, Il1r1, Il27ra, Il5ra, Ngfr, Ccr7, Cxcr5), but produced pro-atherogenic function cytokine (Ifng, Il24, Ltb, Tnfsf4, Tnfsf8, Cxcl12, Ccl22, Ccl4, Ccl5 and Xcl1) in both mice. *ApoE* ^-/-^ induced pro-atherogenic and pro-inflammatory cytokines (Cntf, Il24, Il17c, Cxcl11, Ccl28, Xcl) and receptors (Mpl, Acvr2a, Ccr5) in Ly6C^high^ and Ly6C^low^ MC.

### APC Potential Is Higher in Ly6C^low^ MC and Elevated by *ApoE* ^-/-^ in Ly6C^high^ and Ly6C^low^ MC

**Figure 7:**
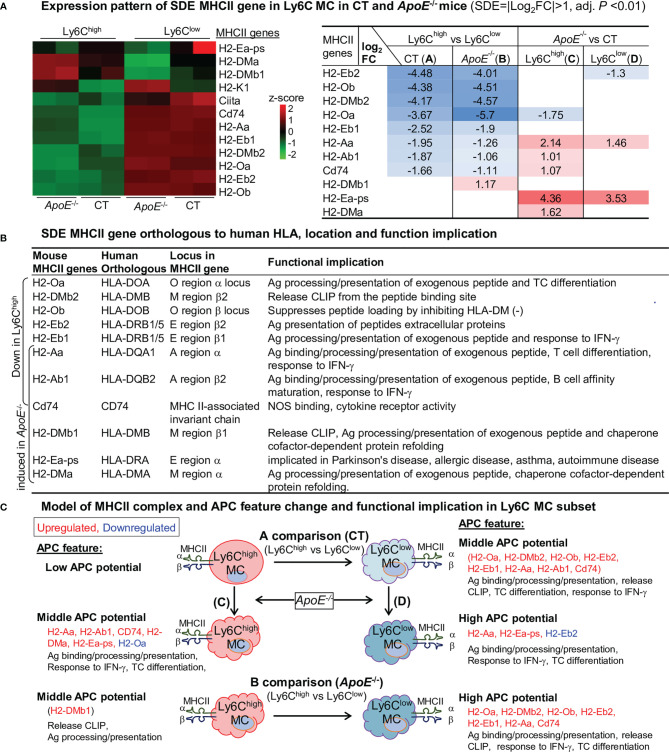
APC potential is higher in Ly6C^low^ MC and elevated by *ApoE* ^-/-^ in Ly6C^low^ and Ly6C^low^ MC. **(A)** Expression pattern of SDE MHCII gene in Ly6C MC in CT and *ApoE* ^-/-^ mice. Heatmap color density indicates the average expression level of a given gene normalized by z-score. Red/blue rainbow background in the table represents the gradient of increased/reduced fold change (log_2_FC>1, log_2_FC<-1). **(B)** SDE MHCII gene orthologous to human HLA, location and function implication. Identified mouse SDE MHCII gene orthologous to human corresponding HLA gene, gene locus and function implication were search from large database (https://www.alliancegenome.org). **(C)** Model of MHCII complex and APC feature and functional implication in Ly6C MC subset. Model outlines that eight SDE MHCII molecules were upregulated in Ly6C^low^ MC in both mice and that *ApoE* ^-/-^ further induced MHCII gene expression. Cloud-like cell shape indicade APC potential. MHCII, major histocompatibility complex class II; Ag, antigen; IFN-γ, Interferon gamma; CLIP, class II-associated invariant chain peptide; NOS, Nitrous Oxide Systems.

Expression profile of 11 SDE MHCII gene are identified through four comparisons (**Figure 7A**). Ly6C^low^ MC is more like APC because of their higher levels of MHCII genes (H2-Oa, H2-DMb2, H2-Ob, H2-Eb2, H2-Eb1, H2-Aa, Cd74) compared to Ly6C^high^ MC in both mice. *ApoE* ^-/-^ elevated the expression levels of MHCII genes in Ly6C^high^ MC (H2-Aa, H2-Ab1, CD74, H2-Dma and H2-Ea-ps) and in Ly6C^low^ MC (H2-Aa and H2-Ea-ps). APC functional feature of SDE MHCII gene is described in **Figure 7B**. We modeled APC features in relevant with MHCII gene change in Ly6C MC subsets in **Figure 7C**.

The Ly6C^high^ MC displayed lower APC potential because of relatively lower levels of all MHCII gene expression. Ly6C^low^ MC had middle APC potential and expressed higher levels of MHCII genes. Most of the MHCII genes in both MC subsets were elevated in *ApoE* ^-/-^ mice. HL promoted Ag binding/processing/presentation, release CLIP in both subsets.

### Summarized Functional Feature of the Innate-Adaptive Immunological Interplay of Ly6C MC in CT and *ApoE* ^-/-^ Mice

**Figure 8:**
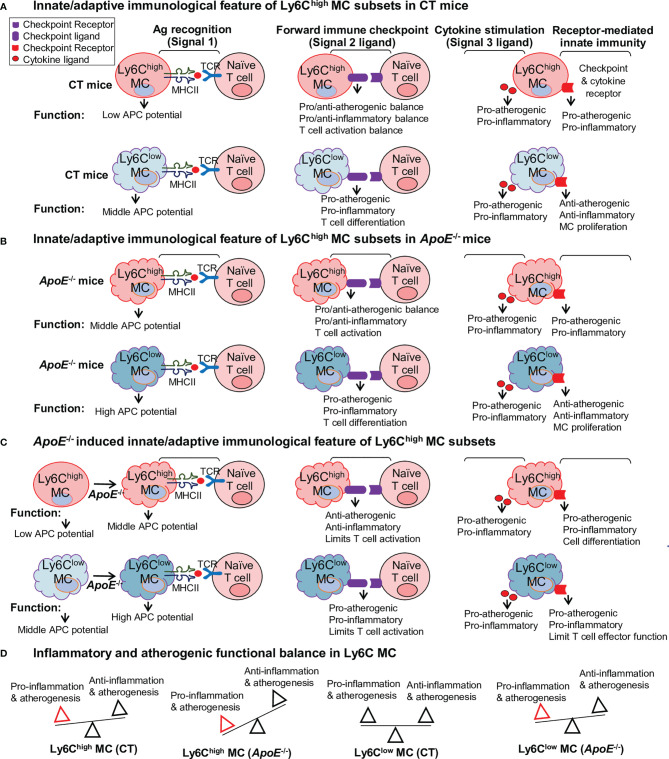
Summarized functional feature of innate-adaptive immunological interplay of Ly6C MC in CT and *ApoE* ^-/-^ mice. Model summarized functional changes derived from molecule signaling presented in **Figures 5**–**7**. **(A)** Innate/adaptive immunological feature of Ly6C^high^ MC subsets in CT mice. **(B)** Innate/adaptive immunological feature of Ly6C^high^ MC subsets in *ApoE* ^-/-^ mice. **(C)** *ApoE* ^-/–^induced innate/adaptive immunological feature of Ly6C^high^ MC subsets. **(D)** Inflammatory and atherogenic functional balance in Ly6C MC. APC, antigen presenting cell; TCR, T cell receptor.

We described the differential functional feature of Ly6C MC subsets in innate-adaptive immunological interplay as 4 aspects: 1. Ag recognition (signal 1), 2. immune checkpoint (signal 2), 3. cytokine stimulation (signal 3), and 4. receptor-mediated innate immune cell regulation (**Figure 8A**). We emphasized APC potential for Ag recognition signal, and focused on 3 function aspects for signals 2 and 3 (pro/anti-atherogenic, pro/anti-inflammatory, MC proliferation and T cell activation) modulated by immune checkpoint and cytokine.

The CT Ly6C^high^ MC presented low APC potential based on their relatively low MHCII molecular expression. Their ligand expression profile implicated a more pro-atherogenic/inflammatory and T cell activation function toward the adaptive immune cells. Their receptor differential expression indicated pro-atherogenic/inflammatory on their own innate immunological function (**Figure 8A**). The CT Ly6C^low^ MC displayed middle APC potential based on their relatively higher MHCII molecular expression.

Their ligand expression profile implicated a more pro-atherogenic/inflammatory and T cell differentiation function toward adaptive immune cell. Differently, the receptor differential expression in CT Ly6C^low^ MC endowed them with anti-atherogenic/inflammatory and MC proliferation function. When comparing Ly6C^high^ MC with Ly6C^low^ MC in the *ApoE* ^-/-^ mice, we found that APC potential was elevated, but other ligand/receptor-determined immunological features were similar as that in CT mice in both MC subsets (**Figure 8B**).

These innate-adaptive immunological functional features were altered by HL condition. *ApoE* ^-/-^ further enhanced APC potential and pro-inflammatory/atherogenic function in both MC subsets when compared with the same MC subset in CT mice (**Figure 8C**). Differently, *ApoE* ^-/-^ limited ligand-related T cell activation in Ly6C^high^ MC and switched the Ly6C^low^ MC from an anti-atherogenic/inflammatory to a pro-atherogenic/inflammatory feature. We modeled pro-inflammatory/atherogenic balance in Ly6C MC subset from *ApoE* ^-/-^ and CT mice in **Figure 8D**. In summary, Ly6C^high^ MC has pro-inflammatory/atherogenic in CT mice. This balance is further inclined towards pro-inflammatory/atherogenic direction in *ApoE* ^-/-^ mice. Ly6C^low^ MC has a balanced inflammatory/atherogenic feature, which is disturbed in *ApoE* ^-/-^ mice and becomes pro-inflammatory/atherogenic.

## Discussion

Inflammatory MC subset differentiation is a major feature in tissue inflammation and atherosclerosis. However, the underlying molecular mechanism remains unclear. This study established transcription profiles of flow cytometry sorted Ly6C^high^ and Ly6C^low^ MC subsets from CT and *ApoE* ^-/-^ mice and explored molecule targets and signaling which potentially determinate immunological features in MC subsets by performing intensive bioinformatic analysis and literature integration. We have 6 major findings: 1) Ly6C^low^ MC engaged more adaptive immune function. 2) Fourteen potential transcriptional axes modulated immunological features in Ly6C MC subset from CT and *ApoE* ^-/-^ mice. 3) Immune checkpoint and cytokine gene profile conferred upon Ly6C^high^ MC pro-inflammatory/atherogenic features and Ly6C^low^ MC anti-inflammatory/atherogenic features. 4) *ApoE* ^-/-^ promoted pro-atherogenic/inflammatory function in both subsets. 5) APC potential is higher in Ly6C^low^ MC and elevated by *ApoE* ^-/-^ in Ly6C^high^ and Ly6C^low^ MC. 6) We established 4 groups of hypothetic molecular signaling models to summarize the regulation of innate and adaptive immunological features in Ly6C MC from both mice. Our findings provided potential regulatory molecular mechanisms in MC subsets delineated innate-adaptive immune response and important guidance for future investigation in this direction.

Previous studies suggested a group of TF regulating MC differentiation from progenitor cells, such as PU.1, CEBPα/β/ε, IRF1/4/8, c-Jun, JunB, STAT1/3 and VDR (7, 8, 23). Transcriptional mechanism has not been clearly addressed for MC subset differentiation. We recently established transcriptome of flow cytometry-sorted Ly6C MC subsets from CT and HHcy *Cbs−/−* mice and discovered 9 upregulated TF (*Cebpa, Cebpd, Cebpe, Irf5/7, Ifi16, Spi1*, and *Stat1/2*) and 6 downregulated TF (*Neurod4, Asb2, Sox5, Pou2af1, Pax5* and *Tbx21*) in Ly6C^high^ MC from CT and HHcy mice (13). We proposed that these 15 TF are potentially involved in Ly6C^high^ (9 TF) and Ly6C^low^ (6 TF) MC differentiation (13). We confirmed that TF CEBPα binds to the *Ly6c* promoter and that its expression was elevated and synergistically increased in HHcy and Type 2 Diabetes Mellitus mice, supported the note that TF CEBPα transactivates *Ly6c* gene and mediates inflammatory MC subset differentiation (3). Others evidence also supported the regulatory role of TF *Irf8* in Ly6C^high^ MC generation using *Irf8−/−* mice, and TF *Cebpb/Irf5 in* Ly6C**^low^** MC generation using *Cebpb* ^-/-^ or *Irf5−/−* mice (24–27).

In this study, we were the first to explore systemic transcriptional regulatory mechanisms in the MC subset in the HL condition. Based on information present in **Figure 4**, we concluded that 12 transcriptional axes potentially modulate immunological features in Ly6C^high^ MC subset. Six trans-activating axis CEBPA-Cxcl10/Il1rn/Csf3r, CEBPD-Ccl2/Csf3r, CEBPE-Il1rn/Csf3r, SPI1-Il1rn/Ccl9/Csf2ra/Csf3r, STAT1-Il15/Cxcl10/Cx3cr1/Csd3r/Fas, IRF5-Cxcl10/Il6/Tnfsf10, IRF7- Il15/Cxcl10/Ccl2, and one trans-suppressing IFI16 -Csf3r may be involved in Ly6C^high^ MC differentiation in CT mice. Two trans-activating axis TBX21-Ifng/Ccl4/Cxcr3/Cxcr5/Il12rb1/Il12rb2/Il2rb/Il7r/Il18rap and Pax5-Cd274/Csf2ra, and one dual-functional TF Myc-Sema4a↑/Tnfrsf9↓/Fas↑/Il5ra↓/Il17rb↓ are potentially involved in Ly6C^low^ MC differentiation. Interestingly, *ApoE* ^-/-^ induced IRF3 by 2-folds which was associated with a 3-folds induction of its downstream target *Il6*.

*ApoE* ^-/-^ mice develop HL similar as that in humans and are an ideal model for atherosclerosis research. We and others reported that inflammatory Ly6C^middle+high^ MC subsets were elevated in early and advanced atherosclerosis lesion, which promoted vascular inflammation (3, 4, 17). Transcriptional molecular processes identified in this study provide models for future investigation.

Very limited information is available to review mechanisms underlying MC subset differentiation. It was suggested that PKCθ may suppress Ly6C^low^ MC in *ApoE* ^-/-^ mice (28), and that cell-intrinsic Notch2 and TLR7-Myd88 pathways may promote Ly6C^low^ MC development from Ly6C^high^ MC under inflammatory conditions (29). Our laboratory is the first to systemically investigate signal pathways mediating MC subset differentiation. In this study, we established a model to summarize top canonical pathways involved in Ly6C MC subset differentiation and responded to *ApoE* ^-/-^ identified by using two bioinformatics tools (IPA and GSEA) (**Figure 3G**). Four pathways (PD-1/PD-L1 checkpoint, paxillin, Rac and OXPHOS) are activated in Ly6C^high^ MC from both *ApoE* ^-/-^ and CT mice, and are potentially involved in Ly6C^high^ MC differentiation. Five pathways (Th1, PKCθ, calcium-induced T cell apoptosis, iCOS/iCOSL signaling in Th cells and Ag-activated B cell receptor to 2^nd^ messenger) are activated in Ly6C^low^ MC from both mice, and may contribute to Ly6C^low^ MC differentiation. In addition, *ApoE* ^-/-^ activated 5 pathways (apelin muscle, PKCθ in T cell, notch, angiopoietin signaling and NFAT regulation) in Ly6C^high^ MC, and 5 pathways (integrin, interferon, crosstalk between DC and NK cells, colorectal cancer metastasis and ephrin receptor) in ly6C^low^ MC, which may be responsible for subsequent function changes.

Recently, through similarly transcriptome analysis approaches, we demonstrated that interferon, inflammasome and PD-1/PD-L1 checkpoint pathways were activated in Ly6C^high^ MC, and calcium-induced T cell apoptosis and iCOS/iCOSL signaling in Th cells were activated in Ly6C^low^ MC from both CT and HHcy *Cbs* ^-/-^ mice, and that *Cbs* ^-/-^ activated NK cell signaling in Ly6C^high^ and suppressed xenobiotic metabolism and melatonin degradation in Ly6C^low^ MC (13). Thus far, we identified 3 canonical pathways shared by Ly6C MC subset from CT, *ApoE* ^-/-^, and *Cbs* ^-/-^ mice. These include PD-1/PD-L1 checkpoint pathway activation in Ly6C^high^ MC, calcium-induced T cell apoptosis and iCOS/iCOSL signaling activation in Ly6C^low^ MC from all three mouse lines.

Interestingly, we found that IFN-α/β signaling was activated in Ly6C^high^ MC from CT mice, and also activated by *ApoE* ^-/-^ in Ly6C^low^ MC suggesting that IFN-α/β signaling represent inflammatory feature in CT Ly6C^high^ and *ApoE* ^-/-^ Ly6C^low^ MC. We also observed a downregulated Ag-activated B cell receptor to 2^nd^ messenger pathway in Ly6C^high^ MC from CT and *ApoE* ^-/-^ mice, supporting the hypothesis that Ly6C^high^ MC has a lower potential for adaptive immune cell activation.

It is known that MC enables adaptive immunity through its APC potential (9). It was reported that circulating Ly6C^high^ MC, and not LY6C^low^ MC, can enter tissues and differentiate into MHCII^+^ MC through interaction with the endothelium (15). Enhanced expression of MHC class II in the Ly6C^high^ MC was associated with inflammatory MC differentiation into mature APC that can activate T cells (30). In the experimental model of multiple sclerosis, Ly6C^high^ MC migrated into the central nervous system and further differentiated into APC during disease progression (31). Despite the above-mentioned studies, the APC potential and regulatory mechanism of MC subsets have not been systemically investigated. This study, for the first time, demonstrated that the Ly6C^high^ MC displayed lower APC potential because of relatively lower levels of all MHCII gene expression, that Ly6C^low^ MC had middle APC potential and expressed higher levels of MHCII genes, and that *ApoE* ^-/-^ elevated most of MHCII genes in both MC subsets. We hypothesize that the higher APC potential in Ly6C^low^ MC may ignite protective adaptive immune response and that *ApoE* ^-/-^ elevated APC potential in both MC subsets may be related to inflammatory response.

The immune checkpoint is a delicate process that controls the direction towards immune suppression or activation. It was reported that LIGHT enhanced proliferating Ly6C^high^ MC and increased atherosclerosis lesion size in *ApoE* ^-/-^Irs2^+/-^HL^-/-^ mice (32). Our current study displayed a systemic expression profile of immune checkpoints in the MC subset and explored their potential role in regulating atherosclerosis based on literature information. We proposed that elevated co-stimulatory checkpoint receptor LIGHT expression in Ly6C^high^ MC may lead to pro-inflammatory/atherogenic reaction, that elevated co-stimulatory checkpoint ligand (CD30L and OX40L), co-inhibitory checkpoint ligand (BTLA and CD160) in Ly6C^low^ MC may induce pro-atherogenic function, and that elevated co-stimulatory checkpoint receptor (GITR and TIM1) and co-inhibitory (CTLA4 and CD96) may contribute to anti-atherogenic function in Ly6C^low^ MC.

MC and MΦ can secrete inflammatory or anti-inflammatory cytokines which play a critical role in the pathogenesis. We found that elevated cytokines (Cxcl10, Ccl2, Tnfsf14, Il1rn, Ccl9, and Cxcl2), and cytokine receptors (Ccr2, Csf3r, Il13ra1, Il3ra, Ltbr, and Acvr1) expression in Ly6C^high^ MC may be associated with their pro-inflammatory/atherogenic function. In contrast, in Ly6C^low^ MC, elevated cytokine receptors (Il12rb2, Il1r1, Il27ra, Il5ra, Ngfr, Ccr7 and Cxcr5) may be related to their anti-inflammatory/atherogenic function toward MC. In addition, elevated expression of pro-inflammatory/atherogenic cytokine ligand (Ifng, Il24, Ltb, Tnfsf4, Tnfsf8, Cxcl12, Ccl22, Ccl4, Ccl5 and Xcl1) in Ly6C^low^ MC may influence immunological balance in other cells. Consistent with our finding, CXCL10 was shown essential for MC pro-inflammatory function (33). *ApoE^-/-^Cxcl10^-/-^* mice presented increased aortic size and a higher incidence of death due to aortic rupture (34). Many studies in both humans and animals have shown the importance of MC chemoattractant protein-1 (MCP-1, also named CCL2) and its receptor CCR2 in pathologies, such as atherosclerosis (35). the CC genotype of MCP-1 SNP rs2857656, independently or in combination with CCR2 V64I genotype, is associated with a high prevalence of carotid artery plaque (36).

Interestingly, we found that *ApoE* ^-/-^ elevated pro-inflammatory Ly6C^high^ MC and reduced anti-inflammatory Ly6C^low^ MC subsets, and *ApoE* ^-/-^ promoted Ly6C^high^ and Ly6C^low^ MC to express higher levels of additional pro-atherogenic and pro-inflammatory cytokines (Cxcl11, Cntf, Il24, and Xcl1) and receptor (Ccr5, Mpl, and Acvr2a). These might be the molecular bases underlying HL-induced atherosclerosis. Pro-inflammatory chemokines CXCL11 was increased in patients with severe transplant coronary artery disease (37). CCR5 has been suggested as a predictor of atherosclerosis progression (38). A CCR5 antagonist (111In-DOTA-DAPTA) was tested as an inflammation imaging tracer for atherosclerosis in mice (39). Bone morphogenetic proteins receptor Acvr2a was reported to induce osteogenic differentiation and MC infiltration in atherosclerosis (40). Mouse MC subset cytokine transcriptome profile provided systemic molecular details and a potential mechanism for MC-related inflammatory reaction.

The current study provided multiple insightful molecular signaling model system for immunological features responsible for pro-inflammatory/atherogenic or anti-inflammatory function of different MC subset. However, future studies are needed to validate these molecular signature and signal pathway changes and to confirm their role in disease condition. The experimental condition of using 20 weeks old *ApoE* ^-/-^ mice fed a high-fat diet for 12 weeks may middle stage of atherosclerotic condition. Immunological molecular change may alter in earlier or late stage of atherosclerosis advanced lesion at different time points.

## Conclusion

Ly6C^high^ MC presented pro-inflammatory/atherogenic features and lower APC potential. Ly6C^low^ MC displayed anti-inflammatory/atherogenic features and higher APC potential. *ApoE* ^-/-^ confers upon both subsets with augmented pro-atherogenic/inflammatory function and APC potential. Our study presented the expression profile of immunological genes in MC subsets, and shaded light on new strategies for the blocking of key immunological genes and signaling as therapeutic targets for treating inflammatory diseases, including atherosclerosis.

## Data Availability Statement

The datasets presented in this study can be found in online repositories. The names of the repository/repositories and accession number(s) can be found below: https://www.ncbi.nlm.nih.gov/geo/GSE189097.

## Ethics Statement

The animal study was reviewed and approved by Temple University, Institutional Animal Care & Use Committee.

## Author Contributions

PY analyzed the data, conceived all figures, and prepared manuscript. QW afforded strong intellectual, data analysis and manuscript support. LS participated in some part of data analysis and manuscript preparation. PF isolated MC subsets from mice, designed RNA-Seq analysis, and provided editing assistance. LL conducted the bioinformatics analyses. J-YP and XQ participated in some of data analysis and provided editing assistance. XY provided strong intellectual and data analysis support. HW designed the study, supervised the project, and prepared the manuscript. All authors contributed to the article and approved the submitted version.

## Funding

This work was supported in part by the National Institutes of Health (NIH) grants HL82774, HL-110764, HL130233, HL131460, DK104114, DK113775, and HL131460 to HW.

## Conflict of Interest

The authors declare that the research was conducted in the absence of any commercial or financial relationships that could be construed as a potential conflict of interest.

## Publisher’s Note

All claims expressed in this article are solely those of the authors and do not necessarily represent those of their affiliated organizations, or those of the publisher, the editors and the reviewers. Any product that may be evaluated in this article, or claim that may be made by its manufacturer, is not guaranteed or endorsed by the publisher.
