# Supplementary material for: Adaptive Immune Response Signaling Is Suppressed in Ly6Chigh Monocyte but Upregulated in Monocyte Subsets of ApoE -/- Mice — Functional Implication in Atherosclerosis
Source: Front Immunol. 2021 Dec 20;12:809208. doi: 10.3389/fimmu.2021.809208 (PMC8721109; doi:10.3389/fimmu.2021.809208)
Supplement: Supplementary file 1 [file DataSheet_1.pdf]

Research Design and Methods

Flow cytometry and cell sorting — Mouse peripheral blood was collected into 1 ml phosphate-buffered saline (PBS) containing 5μM EDTA in fluorescence-activated cell sorting (FACS) tube. White blood cells (WBC) were isolated by using (Ammonium-Chloride-Potassium) ACK lysing buffer (NH4Cl 0.15 M, KHCO3 10.0 mM, Na2 EDTA 0.1 mM) to lyse red blood cells. WBC from 11 mice were pooled and stained with antibodies against CD11b-Brilliant Violet 421 (myeloid cell marker, 0.25 μg/100 μL, clone M1/70), Ly6G-acticated protein C(APC)/Cy7 (granulocyte marker; 0.25 μg/100 μL, clone 1A8), Ly6C-APC (inflammatory MC marker, 0.25 μg/100 μL, clone HK1.4, BD Pharmingen, San Diego, CA), and subjected for flow cytometry cell sorting. $CD11b^{+}$ $Ly6G^{-}$ $Ly6C^{high}$ and $CD11b^{+}$ $Ly6G^{-}$ $Ly6C^{low}$ MC were sorted on a BD FACSAria III cell sorter. Fluorescent activated cells were analyzed offline with FlowJo software (Tree Star Inc, Ashland, OR, version 10) and compiled using Prism software (GraphPad, version 6). All populations were routinely backgated to verify gating and purity.

RNA-Seq in MC subsets — Flow cytometry sorted $CD11b^{+}Ly6G^{-}Ly6C^{high}$ and $CD11b^{+}Ly6G^{-}Ly6C^{low}$ cells from control and *ApoE*$^{-/-}$ WBC (100,000/MC) were collected in 1400 μL QIAzol Lysis Reagent (Qiagen, Germantown, MD) for total RNA extraction. Samples were quality checked on an Agilent Bioanalyzer 2100 using pico RNA chip for RNA integrity number. Total RNA (50-100 ng/sample) were used for cDNA library construction after ribosomal cDNA depletion using Takara pico-input kit. Pooled samples were run for sequencing analysis in duplicate on NextSeq 500 (CT) and Illumina Hiseq 4000 sequencer (*ApoE*$^{-/-}$). Overall, we obtained around 40 million reads from each sample as the raw data.

RNA-seq data from this study are available from the corresponding author upon reasonable request in reference to recent similar publication [1]. Details for major RNA-seq data resources can be found in Supplementary Material.

RNA-seq data processing — The raw RNA-seq data was analyzed using the statistical computing environment R, the Bioconductor suite of packages for R and RStudio (tidyverse, reshape2, tximport, biomaRt, RColorBrewer, genefilter, edgeR, matrixStats, hrbrthemes, gplots, limma, DT, gt, plotly, beepr, skimr, cowplot, data.table, sva). The raw reads were mapped to the mouse reference transcriptome (mouse cDNA

FASTA from ensembl, website http://uswest.ensembl.org/info/data/ftp/index.html) using Kallisto, version 0.45. Genes with less than 1 count per million reads in at least 2 or more samples were filtered out. This reduced the number of genes to 16,476 normalized genes.

Principle components analysis (PCA) — PCA was performed to examine the variance of RNA-seq data. RNA-seq data from control and *ApoE*$^{-/-}$ mice were produced at different times and processed to remove batch effects and other unwanted noise using ComBat approach [2, 3]. The first 2 principal components (PC1 and PC2) were used to depict the similarity between samples.

Identification of significantly differentially expressed (SDE) gene — SDE genes were identified using the Bioconductor suite of Limma packages in RStudio software with the criteria of $|\text{Log}_2$ fold change (FC)$|$ more than 1 (FC>2) and adjusted *P*-value less than 0.01. We identified genes differentially expressed ($|FC|>2, P<0.01$) in Ly6C^{high} and Ly6C^{low} MC by performing four pairs of comparisons: **A**. Ly6C^{high} vs Ly6C^{low} (control), **B**. Ly6C^{high} vs Ly6C^{low} (*ApoE*$^{-/-}$), **C**. *ApoE*$^{-/-}$ vs control (Ly6C^{high}), **D**. *ApoE*$^{-/-}$ vs control (Ly6C^{low}). We identified MHCII, cytokine and immune checkpoint gene based on the current literature [4, 5]. SDE immunological genes were overlapped with SDE gene in immunological gene.

Heatmap —Heatmap was generated in RStudio using the pheatmap package to present the expression levels of SDE genes. The color density in the heatmap indicates the average expression levels of a given gene normalized by z-score.

Identification of functional pathways — We used Ingenuity Pathway Analysis (IPA) version 7.1 (IPA, Ingenuity Systems, https://www.ingenuity.com) to identify functional pathways. SDE genes were identified and uploaded into IPA for analysis. The general canonical functional pathways were established for SDE genes identified in above mentioned 4 comparison groups, as we have previously reported [6, 7].

Overlap analysis of SDE genes and pathways— SDE genes and functional pathways identified from above mentioned 4 comparisons were subjected for overlapping analysis (http://bioinformatics.psb.ugent.be/webtools/Venn/). Venn diagrams were displayed to present SDE genes and pathways overlaps between comparisons.

Identification of transcriptional signaling — We identified SDE TFs and matched with their corresponding SDE immunological genes by referencing TF-matched gene sets using IPA upstream analysis. The significate matches were

recognized as potential transcriptional signaling (TF/targeted molecule axis) based on p-values < 0.01, $|z\text{-scores}|>2$, calculated by using Fisher's Exact Test.

References

1. Kimball A, Schaller M, Joshi A, Davis FM, denDekker A, Boniakowski A, Bermick J, Obi A, Moore B, Henke PK, Kunkel SL, Gallagher KA. Ly6c(hi) blood monocyte/macrophage drive chronic inflammation and impair wound healing in diabetes mellitus. *Arteriosclerosis, thrombosis, and vascular biology*. 2018;38:1102-1114
2. Haghverdi L, Lun ATL, Morgan MD, Marioni JC. Batch effects in single-cell rna-sequencing data are corrected by matching mutual nearest neighbors. *Nature biotechnology*. 2018;36:421-427
3. Johnson EN, Burns TC, Hayda RA, Hospenthal DR, Murray CK. Infectious complications of open type iii tibial fractures among combat casualties. *Clinical infectious diseases : an official publication of the Infectious Diseases Society of America*. 2007;45:409-415
4. Zhang R, Saredy J, Shao Y, Yao T, Liu L, Saaoud F, Yang WY, Sun Y, Johnson C, Drummer Ct, Fu H, Lu Y, Xu K, Liu M, Wang J, Cutler E, Yu D, Jiang X, Li Y, Li R, Wang L, Choi ET, Wang H, Yang X. End-stage renal disease is different from chronic kidney disease in upregulating ros-modulated proinflammatory secretome in pbmcs - a novel multiple-hit model for disease progression. *Redox biology*. 2020;34:101460
5. Dai J, Fang P, Saredy J, Xi H, Ramon C, Yang W, Choi ET, Ji Y, Mao W, Yang X, Wang H. Metabolism-associated danger signal-induced immune response and reverse immune checkpoint-activated cd40(+) monocyte differentiation. *Journal of hematology & oncology*. 2017;10:141
6. Li YF, Nanayakkara G, Sun Y, Li X, Wang L, Cueto R, Shao Y, Fu H, Johnson C, Cheng J, Chen X, Hu W, Yu J, Choi ET, Wang H, Yang XF. Analyses of caspase-1-regulated transcriptomes in various tissues lead to identification of novel il-1beta-, il-18- and sirtuin-1-independent pathways. *Journal of hematology & oncology*. 2017;10:40
7. Wang L, Fu H, Nanayakkara G, Li Y, Shao Y, Johnson C, Cheng J, Yang WY, Yang F, Lavallee M, Xu Y, Cheng X, Xi H, Yi J, Yu J, Choi ET, Wang H, Yang X. Novel extracellular and nuclear caspase-1 and inflammasomes propagate inflammation and regulate gene expression: A comprehensive database mining study. *Journal of hematology & oncology*. 2016;9:122
