# Supplementary material for: Adaptive Immune Response Signaling Is Suppressed in Ly6Chigh Monocyte but Upregulated in Monocyte Subsets of ApoE -/- Mice — Functional Implication in Atherosclerosis
Source: Front Immunol. 2021 Dec 20;12:809208. doi: 10.3389/fimmu.2021.809208 (PMC8721109; doi:10.3389/fimmu.2021.809208)
Supplement: Supplementary file 2 [file DataSheet_2.pdf]

Adaptive immune response signaling is suppressed in Ly6C^{high} monocyte but upregulated in monocyte subsets of *ApoE*$^{-/-}$ mice — functional implication in atherosclerosis

Pingping Yang[1,2#], Qinghua Wu[1#], Lizhe Sun[2,3], Pu Fang[2], Lu Liu[2], Yong Ji[4], Joon-Young Park, Xuebin Qin[5], Xiaofeng Yang[2], Hong Wang[2*]

[1]Departments of Cardiovascular Medicine and Endocrinology, the Second Affiliated Hospital of Nanchang University, Nanchang, Jiangxi, 330006, People's Republic of China

[2]Center for Metabolic Disease Research, Department of Cardiovascular science, Lewis Kats School of Medicine, Temple University, Philadelphia, PA, USA

[3]Department of Cardiovascular Medicine, the First Affiliated Hospital of Xi'an Jiaotong University, Xi'an 710061, People's Republic of China

[4]Key Laboratory of Cardiovascular Disease and Molecular Intervention, Nanjing Medical University, Nanjing, People's Republic of China

[5]Tulane National Primate Research Center, School of Medicine, Tulane University, Covington, LA, USA

#Yang P and Wu Q contributed equally to this work

*Correspondence should to Professor Hong Wang (Ph.D. & M.D.); Center for Metabolic Disease Research, Lewis Kats School of Medicine, Medical Education and Research Building, Room 1060, 3500 N Broad St, Philadelphia, PA 19140 USA; E-mail: hong.wang@temple.edu

A. Gating and sorting strategy to isolate Ly6C^{high} and Ly6C^{low} MC in CT and *ApoE$^{-/-}$* mice

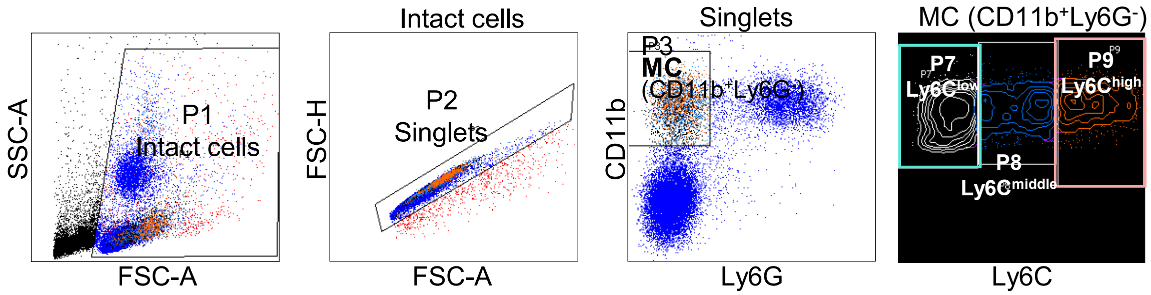

Tube: ***ApoE$^{-/-}$***

RAN 2.3e6 TOAL CELLS; SORTED 100,000 P3; P7 P8; P9

Population	#Events	%Parent	%Total
All Events	30,000	####	100.0
P1	20,449	68.2	68.2
P2	19,808	96.9	66.0
P3	4,066	20.5	13.6
P7	1,312	32.3	4.4
P8	964	23.7	3.2
P9	1,776	43.7	5.9

Tube: **CT**

RAN 3.6e6 TOAL CELLS; SORTED 100,000 P3; P7 P8; P9

Population	#Events	%Parent	%Total
All Events	30,000	####	100.0
P1	21,827	72.8	72.8
P2	21,314	97.6	71.0
P3	3,315	15.6	11.0
P7	1,193	36.0	4.0
P8	1,098	33.1	3.7
P9	1,006	30.3	3.4

B. MC subset cell yield from peripheral blood by cell sorting

P#	Cell subset (%)	*ApoE$^{-/-}$* (%)	CT (%)
P3	MC (MC/live cell %)	100,000 (19.8)	100,000 (15.5)
P7	Ly6C^{low} MC (Ly6C^{low} MC/total MC%)	32,300 (32.3)	36,000 (36.0)
P8	Ly6C^{middle} MC (Ly6C^{middle} MC/total MC %)	23,700 (23.7)	33,100 (33.1)
P9	Ly6C^{high}MC (Ly6C^{high}MC/MC %)	43,700 (43.7)	30,300 (30.3)

Supplementary figure 1. A. Gating and sorting strategy to isolate Ly6C^{high} and Ly6C^{low} MC in CT and *ApoE$^{-/-}$* mice. Mouse white blood cell were prepared from peripheral blood and stained with antibody against CD11b, Ly6G and Ly6C and subjected for flow cytometry cell sorting. Intact cells (P1, 68.2% in *ApoE$^{-/-}$*, 72.8% in CT) were recognized based on higher FSC-A (larger size). Singlets (P2, 66.0% in *ApoE$^{-/-}$*, 71.0% in CT) were identified by using FSC-H versus FSC-A appeared on a diagonal. CD11b^{+}Ly6G^{-} cells (P3, 13.6% in *ApoE$^{-/-}$*, 11.0% in CT) were selected as MC. MC subsets (P9 CD11b^{+}Ly6G^{-}Ly6C^{high}, and P7 CD11b^{+}Ly6G^{-}Ly6C^{low}) were sorted based on Ly6C levels. The quantification of MC was used flow cytometry analysis for Ly6C^{high} and Ly6C^{low} MC in CT and *ApoE$^{-/-}$*. **B. MC subset cell yield from peripheral blood by cell sorting.** Flow cytometry sorted 100,000 MC with 43,700 Ly6C^{high} and 32,300 Ly6C^{low} MC in *ApoE$^{-/-}$* mice and 30,300 Ly6C^{high} and 36,000 Ly6C^{low} MC in CT mice.

Supplementary table 1A. Ly6C^{high}(CT) vs Ly6C^{low}(CT)

a. Top 20 canonical pathway by top-down analysis (GSEA, |NES|>1)

Pathway name	NES	NOM p-val	FDR q-val
**Interferon α/β signaling**	**2.25**	**0.00**	**0.01**
Oxidative phosphorylation	1.92	0.00	0.28
IL3 pathway	1.85	0.02	0.40
Insulin receptor recycling	1.84	0.00	0.32
Alk1 pathway	1.79	0.00	0.43
Perk regulates gene expression	1.76	0.00	0.44
ATF4 activates genes in response to endoplasmic reticulum stress	1.75	0.01	0.42
Parkinsons disease	1.74	0.00	0.38
LDL clearance	1.70	0.02	0.47
Macroautophagy	1.68	0.00	0.51
Autophagy	1.68	0.00	0.46
Detoxification of reactive oxygen species	1.68	0.01	0.44
Huntingtons disease	1.65	0.00	0.48
Antigen processing cross presentation	1.63	0.00	0.51
HDMS demethylate histones	1.63	0.02	0.50
Beta catenin phosphorylation cascade	1.62	0.05	0.48
Regulation of runx3 expression and activity	1.62	0.00	0.45
Signaling by bmp	1.60	0.03	0.49
Cholesterol biosynthesis	1.60	0.02	0.49
Gmcsf pathway	1.59	0.01	0.50
**Antigen activates BCR leading to generation of 2nd messengers**	**-1.79**	**0.00**	**0.01**
**Gap junction assembly**	**-1.72**	**0.00**	**0.05**
**Gap junction trafficking and regulation**	**-1.66**	**0.00**	**0.14**
**Ncam1 interactions**	**-1.65**	**0.00**	**0.15**
**Carboxyterminal post translational modifications of tubulin**	**-1.64**	**0.00**	**0.15**
**Phase 0 rapid depolarisation**	**-1.62**	**0.01**	**0.19**
**IAT2 NTAL lab on calcium mobilization**	**-1.60**	**0.00**	**0.24**
Potassium channels	-1.59	0.00	0.26
Primary immunodeficiency	-1.58	0.01	0.30
Nkt pathway	-1.54	0.02	0.50
Interleukin 37 signaling	-1.53	0.02	0.53
IL12 2pathway	-1.53	0.01	0.52
Complement cascade	-1.53	0.02	0.49
Ncam signaling for neurite out growth	-1.52	0.01	0.47
IL12 stat4 pathway	-1.52	0.01	0.45
Carm er pathway	-1.51	0.03	0.51
Csk pathway	-1.51	0.02	0.49
Tob1 pathway	-1.51	0.02	0.47
Striated muscle contraction	-1.49	0.02	0.57
Il12 pathway	-1.48	0.03	0.63

Up-regulated Down-regulated

b. Representative SDE genes of top 20 canonical pathways by top-down analysis (GSEA, |LogFC|>1, adj. *P*<0.01)

Up-regulated (40 genes)

Gene	logFC	adj.P	Gene	logFC	adj.P
Plcb1	5.21	0.00	Atf3	2.51	0.00
Ccl2	4.36	0.00	Ifitm3	2.48	0.00
Mx2	3.44	0.00	Oas3	2.44	0.00
Fos	3.32	0.00	Acvrl1	2.38	0.00
Ifit3	3.27	0.00	Isg15	2.34	0.00
Id1	3.21	0.00	Soat1	2.31	0.00
Mx1	3.06	0.00	Ap2a2	2.11	0.00
Cox6b2	3.00	0.00	Mvd	2.05	0.00
Irf7	2.86	0.00	Lsr	2.02	0.00
Npc2	2.84	0.00	Lamtor4	2.01	0.00
Acvr1	2.81	0.00	Atg9b	1.99	0.00
Oas2	2.73	0.00	Eng	1.92	0.00
Prdx5	2.67	0.00	Cd14	1.92	0.00
Ctsl	2.60	0.00	Bst2	1.89	0.00
Ifit2	2.60	0.00	Usp18	1.89	0.00
Gpx3	2.59	0.00	Atp6v1b2	1.88	0.00
Exosc6	2.59	0.00	Frat2	1.86	0.00
Ifit1	2.59	0.00	Samhd1	1.82	0.00
Xaf1	2.54	0.00	Ldlr	1.80	0.00
Acvr2b	2.53	0.00	Stat2	1.80	0.00

Down-regulated (40 genes)

Gene	logFC	adj.P	Gene	logFC	adj.P
Ptpn14	-6.74	0.00	Ttll7	-4.02	0.00
Blk	-5.88	0.00	Kcng1	-4.01	0.00
Cacna1i	-5.71	0.00	Ccr7	-3.83	0.00
Blnk	-5.62	0.00	Ptpn13	-3.77	0.00
Cacna1s	-5.61	0.00	Prf1	-3.76	0.00
Cd19	-5.60	0.00	Ighg1	-3.49	0.00
Cd79a	-5.55	0.00	Gzma	-3.43	0.00
Iglc2	-5.09	0.00	Tbx21	-3.40	0.00
Ighd	-5.06	0.00	Tubb3	-3.35	0.00
Cd55	-4.76	0.00	Gnb3	-3.33	0.00
Cd79b	-4.67	0.00	Ighv1-69	-3.33	0.00
Tnfrsf13c	-4.61	0.00	Eomes	-3.33	0.00
Iglc3	-4.55	0.00	Ccl4	-3.20	0.00
Agbl3	-4.52	0.00	Aicda	-3.08	0.00
Tnnt1	-4.48	0.00	Il18r1	-3.06	0.00
Kcnk10	-4.37	0.00	Ccl3	-3.04	0.00
Cd22	-4.34	0.00	Gzmb	-2.99	0.00
Il2ra	-4.23	0.00	Il2rb	-2.91	0.00
Ifng	-4.14	0.00	Grip1	-2.91	0.01
Il12rb1	-4.08	0.00	Tubb2b	-2.84	0.00

Supplementary table 1. Identification of pathways and their top SDE genes in four pairs of comparisons. A. Ly6C^{high} vs Ly6C^{low} (CT); B. Ly6C^{high} vs Ly6C^{low}(*ApoE*$^{-/-}$); C. *ApoE*$^{-/-}$ vs CT (Ly6C^{high}); D. *ApoE*$^{-/-}$ vs CT (Ly6C^{low}). Top canonical pathways were identified by top-down analysis using GSEA and IPA software. All SDE genes involved in these top pathways are listed. **(a). Top 20 up/down regulated GSEA pathway** (GSEA, |NES|>1)**.** GSEA results showing pathways enriched in the top 20 upregulated or downregulated pathways with the criteria of |NES|>1. The most enriched significant pathways from GSEA using threshold of |NES|>1.5, FDR < 0.25, and nominal p value<0.01 are marked using different color (red=up-regulated, blue=down-regulated. **(b). Representative top 40 up/down SDE genes from IPA canonical pathways analysis**. The top 40 up- and down-regulated SDE genes are identified from all IPA pathways in (**iii**) using the criteria of |Log_2FC| more than 1 (=two-fold change) and adjusted P value less than 0.01. Molecule type is classified according to the IPA system definition. Molecule type for immunological gene sets is defined based on our previous publication (PMID: 32179051). **Abbreviations:** MC, monocyte; MΦ, macrophage; TREM1, The triggering receptor expressed on myeloid cells 1; GPCRs, G-protein-coupled receptors; PFKFB4, 6-phosphofructo-2-kinase/fructose-2,6-biphosphatase 4; GSEA, Gene Set Enrichment Analysis; ES, enrichment score; NES, Normalized enrichment score.; FDR, false discovery rate. BCR, B cell receptor. **Abbreviation for** gene names refer to list in website https://www.genecards.org/.

Supplementary table 1B. Ly6C^{high}(*ApoE$^{-/-}$*) vs Ly6C^{low}(*ApoE$^{-/-}$*)

a. Top 20 up/down regulated pathway (GSEA, |NES|>1)

Pathway name	NES	NOM p-val	FDR q-val
Parkinsons disease	1.81	0.00	0.56
Oxidative phosphorylation	1.81	0.00	0.30
**Formation of fibrin clot clotting cascade**	**1.79**	**0.01**	**0.24**
VDR pathway	1.74	0.01	0.34
Endogenous pathway	1.71	0.00	0.39
Synaptic adhesion like molecules	1.68	0.01	0.42
LDL clearance	1.65	0.02	0.47
Glyoxylate metabolism and glycine degradation	1.59	0.01	0.72
IL6 pathway	1.58	0.02	0.70
Drug metabolism cytochrome p450	1.55	0.03	0.82
Gcr pathway	1.54	0.04	0.77
Neutrophil degranulation	1.53	0.00	0.75
Insulin receptor recycling	1.52	0.02	0.75
Pdzs pathway	1.50	0.04	0.84
Notch4 intracellular domain regulates transcription	1.48	0.07	0.85
Alk1 pathway	1.48	0.03	0.82
Endosomal sorting complex required for transport escrt	1.46	0.04	0.88
Signaling by notch3	1.46	0.02	0.83
Drug metabolism other enzymes	1.45	0.07	0.82
Glycosphingolipid metabolism	1.45	0.03	0.79
**Antigen activates BCR leading to generation of 2nd messengers**	**-1.71**	**0.00**	**0.16**
**Role of LAT2 NTAL lab on calcium mobilization**	**-1.67**	**0.00**	**0.20**
**Phase 0 rapid depolarisation**	**-1.66**	**0.00**	**0.16**
**Fceri mediated caplus2 mobilization**	**-1.65**	**0.00**	**0.15**
**Primary immunodeficiency**	**-1.63**	**0.00**	**0.22**
Activation of ampk downstream of nmdars	-1.61	0.00	0.26
Inwardly rectifying kplus channels	-1.60	0.00	0.26
G protein gated potassium channels	-1.58	0.01	0.34
NKT pathway	-1.55	0.01	0.49
Initial triggering of complement	-1.54	0.01	0.53
IL12 2pathway	-1.54	0.00	0.50
Formation of tubulin folding intermediates by cct tric	-1.54	0.01	0.47
Gap junction assembly	-1.54	0.01	0.43
IL2 stat5 pathway	-1.54	0.01	0.43
Ctla4 pathway	-1.52	0.01	0.49
Rho gtpases activate iqgaps	-1.52	0.01	0.51
LIS1 pathway	-1.52	0.02	0.48
Potassium channels	-1.51	0.01	0.47
Chrebp pathway	-1.49	0.03	0.59
Gap junction trafficking and regulation	-1.49	0.02	0.56

Up-regulated Down-regulated

b. SDE gene list of top 40 up/down regulated gene from GSEA pathways (SDE=|LogFC|>1, adj. *P*<0.01, Identified 146 up regulated and 85 down regulated SDE, only shown top 40 in each groups)

Up-regulated (40 genes)

Gene	logFC	adj.P	Gene	logFC	adj.P
Clec5a	7.24	0.00	Id1	2.97	0.00
Vcan	5.93	0.00	Cd33	2.92	0.00
Cd177	5.78	0.00	Mgam	2.86	0.01
Mmp8	5.60	0.00	Mgst1	2.79	0.00
F13a1	5.23	0.00	Acta2	2.78	0.00
Slpi	4.98	0.00	C3	2.78	0.00
Ptprf	4.97	0.00	Stbd1	2.78	0.00
Tarm1	4.43	0.00	Lcn2	2.77	0.00
Rab44	4.28	0.00	Gria3	2.77	0.01
Il6	4.27	0.00	Bst2	2.75	0.00
Prtn3	4.12	0.00	S100a9	2.73	0.00
Lrg1	3.85	0.00	Svip	2.66	0.00
Cd93	3.82	0.00	Anxa1	2.58	0.00
Fos	3.72	0.00	Pnp	2.47	0.00
Maob	3.71	0.00	Ugt1a5	2.43	0.00
Bst1	3.62	0.00	F10	2.42	0.00
Ugt1a1	3.59	0.00	C3ar1	2.37	0.00
Acvrl1	3.57	0.00	Lsr	2.36	0.00
Iqgap2	3.09	0.00	Eng	2.35	0.00
Serpinb10	3.05	0.00	Npc2	2.34	0.00

Down-regulated (40 genes)

Gene	logFC	adj.P	Gene	logFC	adj.P
Kcnq4	-6.76	0.00	Lta	-3.95	0.00
Zap70	-6.44	0.00	Il2rb	-3.81	0.00
Cacna1s	-6.26	0.00	Ighd	-3.79	0.00
Kcns1	-5.99	0.00	Tjp1	-3.73	0.01
Blk	-5.87	0.00	Tubb2b	-3.69	0.00
Cd19	-5.48	0.00	Prf1	-3.68	0.00
Fgf13	-5.30	0.00	Kcng1	-3.68	0.00
Iglc2	-5.08	0.00	Gzmb	-3.62	0.00
Cd79a	-5.01	0.00	Il12a	-3.61	0.00
Ighv1-69	-4.92	0.00	Gzma	-3.61	0.00
Iglc3	-4.89	0.00	Ccr7	-3.60	0.00
Ctla4	-4.52	0.00	Scn4a	-3.59	0.00
Blnk	-4.38	0.00	Lck	-3.57	0.00
Cdk5r1	-4.29	0.00	Icos	-3.51	0.00
Ighv2-5	-4.25	0.00	Eomes	-3.50	0.00
Gnb3	-4.15	0.00	Myc	-3.47	0.00
Cd79b	-4.13	0.00	Tnfrsf13c	-3.44	0.00
Ccnd2	-4.01	0.00	Ifng	-3.37	0.00
Gabbr1	-4.00	0.00	Kcnk13	-3.34	0.00
Cd22	-3.99	0.00	Ccl4	-3.11	0.00

Supplementary table 1C. Ly6C^{high}(*ApoE*$^{-/-}$) vs Ly6C^{high}(CT)

A. Top 20 up/down regulated pathway (GSEA, |NES|>1)

Pathway name	NES	NOM p-val	FDR q-val
Gene and protein expression by jak stat signaling after interleukin 12 stimulation	1.75	0.00	0.66
Hes hey pathway	1.71	0.00	0.59
Protein export	1.60	0.01	1.00
Arenrf2 pathway	1.60	0.02	1.00
Insulin processing	1.55	0.02	1.00
Cell extracellular matrix interactions	1.54	0.02	1.00
Shp2 pathway	1.52	0.02	1.00
G1 phase	1.52	0.02	1.00
Nos1 pathway	1.52	0.02	1.00
Histidine metabolism	1.51	0.04	1.00
Proximal tubule bicarbonate reclamation	1.51	0.05	1.00
Regulation of the actin cytoskeleton by rho gtpases	1.51	0.04	1.00
Nfkappab atypical pathway	1.49	0.06	1.00
Phenylalanine metabolism	1.48	0.05	1.00
Tyrosine metabolism	1.46	0.05	1.00
Cysteine and methionine metabolism	1.45	0.03	1.00
Interleukin 6 family signaling	1.45	0.06	1.00
Vegfr2 mediated cell proliferation	1.44	0.06	1.00
Beta alanine metabolism	1.44	0.07	1.00
Vegf pathway	1.43	0.06	1.00
G2 pathway	-1.78	0.00	0.45
**ATRBRCA pathway**	**-1.77**	**0.00**	**0.25**
ATM pathway	-1.71	0.01	0.51
Homologous dna pairing and strand exchange	-1.70	0.00	0.44
Hdr through homologous recombination hrr	-1.67	0.00	0.49
Resolution of d loop structures through synthesis dependent strand annealing sdsa	-1.67	0.00	0.43
Resolution of d loop structures	-1.66	0.01	0.43
Bard1 pathway	-1.65	0.01	0.42
Bladder cancer	-1.63	0.02	0.51
Interleukin 7 signaling	-1.62	0.01	0.49
Piwi interacting rna pirna biogenesis	-1.61	0.01	0.53
Il7 pathway	-1.59	0.02	0.58
Aldosterone regulated sodium reabsorption	-1.58	0.02	0.60
Pentose phosphate pathway	-1.58	0.02	0.58
Activation of ampk downstream of nmdars	-1.58	0.02	0.55
Downstream signaling of activated fgfr3	-1.57	0.02	0.58
Signaling by bmp	-1.55	0.03	0.61
Rap1 signalling	-1.51	0.03	0.93
Hif1a pathway	-1.50	0.03	0.93
Insulin glucose pathway	-1.50	0.05	0.88

b. SDE list of top 40 up/down regulated gene from GSEA pathways (SDEs=|LogFC|>1, adj. *P*<0.01, Identified 16 up regulated and 28 down regulated SDE) supplement

Up-regulated (16 genes)

Gene	logFC	adj.P
Serpinb2	5.08	0.00
Gsta2	4.24	0.00
Cd4	3.89	0.00
Il4i1	3.64	0.00
Lif	3.57	0.00
Cdkn1c	3.10	0.00
Ar	2.52	0.00
Slc30a7	2.50	0.00
Fah	2.02	0.00
Itpr3	2.02	0.00
Maff	2.00	0.00
Adi1	1.88	0.00
Il2ra	1.82	0.00
Pcsk2	1.54	0.01
Cndp1	1.48	0.00
Srm	1.32	0.01

Down-regulated (28 genes)

Gene	logFC	adj.P
Bmpr1a	-5.83	0.00
Rapgef3	-5.04	0.01
Fgfr3	-4.91	0.00
Mmp9	-4.83	0.01
Pik3r3	-3.30	0.00
Dapk2	-3.27	0.00
Plcg1	-3.14	0.00
Brca1	-2.85	0.00
Chek1	-2.62	0.00
Tubb2b	-2.58	0.00
Tdrkh	-2.56	0.00
Ccnb1	-2.47	0.00
Slc9a3r2	-2.45	0.00
Il7r	-2.44	0.01
Aldob	-2.26	0.00
Prkag3	-2.25	0.00
Socs1	-2.24	0.00
Aldoc	-2.18	0.00
Egln3	-1.91	0.00
Fstl1	-1.86	0.00
Tbc1d4	-1.68	0.00
Tktl1	-1.63	0.01
Sfn	-1.50	0.00
Akt1	-1.45	0.00
Eme1	-1.39	0.00
Hsd11b2	-1.25	0.00
Fancf	-1.13	0.00
Cul2	-1.10	0.01

Supplementary table 1D. Ly6C^{low}(*ApoE*$^{-/-}$) vs Ly6C^{low}(CT)

a. Top 20 up/down regulated pathway (GSEA, |NES|>1)

Pathway name	NES	NOM p-val	FDR q-val
**Interferon α/β signaling**	**1.90**	**0.00**	**0.12**
P53hypoxia pathway	1.75	0.01	0.78
Interleukin 20 family signaling	1.71	0.02	0.95
Mcalpain pathway	1.70	0.00	0.72
Creb pathway	1.68	0.01	0.77
Integrin a4b1 pathway	1.67	0.01	0.73
Vip pathway	1.62	0.01	0.99
Jak stat signaling pathway	1.62	0.00	0.88
Antiviral mechanism by ifn stimulated genes	1.61	0.00	0.86
Ptp1b pathway	1.59	0.01	0.93
Pi3k plc trk pathway	1.59	0.02	0.89
Ppara pathway	1.56	0.02	1.00
S1p s1p3 pathway	1.56	0.02	1.00
Ret pathway	1.55	0.02	0.97
Transcription of G2F targets under negative control by dream complex	1.54	0.03	0.98
Activation of irf3 irf7 mediated by tbk1 ikk epsilon	1.54	0.03	0.96
Glutathione metabolism	1.51	0.04	1.00
S1p meta pathway	1.50	0.04	1.00
Downstream signal transduction	1.50	0.04	1.00
Igf1r pathway	1.47	0.05	1.00
Tyrosine metabolism	-1.72	0.00	0.33
**Rho GTPases activate NADPH oxidases**	**-1.70**	**0.00**	**0.22**
Toll endogenous pathway	-1.64	0.01	0.58
Histidine metabolism	-1.62	0.01	0.57
Notch4 intracellular domain regulates transcription	-1.61	0.00	0.53
Hedgehog on state	-1.61	0.00	0.44
Signaling by notch2	-1.61	0.00	0.39
Plasma lipoprotein clearance	-1.60	0.00	0.40
Phenylalanine metabolism	-1.59	0.00	0.40
Tap63 pathway	-1.59	0.00	0.36
Tgfb pathway	-1.55	0.01	0.59
Glycine serine and threonine metabolism	-1.53	0.01	0.65
Trafficking of glur2 containing ampa receptors	-1.53	0.02	0.68
Negative regulation of met activity	-1.52	0.02	0.63
Tp53 regulates transcription of additional cell cycle genes whose exact role in the p53 pathway rema	-1.52	0.02	0.65
Notch pathway	-1.52	0.01	0.61
Her2 pathway	-1.50	0.02	0.71
Progesterone mediated oocyte maturation	-1.50	0.01	0.68
Igf1 pathway	-1.49	0.04	0.69
Butanoate metabolism	-1.49	0.03	0.66

b. SDE list of top 40 up/down regulated gene from GSEA pathways (SDEs=|LogFC|>1, adj. *P*<0.01, Identified 31 up regulated and 48 down regulated SDE, only shown top 40 in down regulated groups)

Up-Gene	logFC	adj.P
Gab1	4.55	0.00
Isg20	4.05	0.00
Gsta2	3.86	0.00
Cntf	3.81	0.00
Gna14	3.70	0.00
Ifitm1	3.06	0.00
Il10	3.00	0.00
Rsad2	2.90	0.00
Il20rb	2.74	0.00
Mpl	2.33	0.00
Gstm1	2.29	0.00
Oas2	2.16	0.00
Isg15	2.12	0.00
Oas3	2.07	0.00
Capns2	2.04	0.00
Il12a	1.89	0.00
Kpna2	1.84	0.00
Gclm	1.82	0.00
Eif4e3	1.79	0.00
Gdnf	1.71	0.00
Tfdp2	1.64	0.00
Cd14	1.59	0.00
Nr1h3	1.52	0.00
Egr1	1.52	0.00
Ube2l6	1.45	0.00
Ifi27	1.32	0.00
Itgb3	1.28	0.00
Il15ra	1.23	0.00
Gna11	1.16	0.00
Ccnd1	1.06	0.00
Gadd45a	1.03	0.00

Down-Gene	logFC	adj.P
ApoE	-6.78	0.00
Aldh5a1	-5.64	0.00
Apob	-5.47	0.00
Cables1	-5.44	0.00
Tat	-5.03	0.00
Sardh	-4.77	0.00
Il6	-4.71	0.00
S100a9	-4.44	0.00
Cdc25c	-4.40	0.00
Gli1	-4.20	0.00
Jag2	-4.18	0.00
Igf1	-4.09	0.00
Vldlr	-3.99	0.00
Bgn	-3.70	0.00
Bud23	-3.28	0.00
Kif7	-3.26	0.00
Nox1	-3.09	0.00
Mapk13	-3.00	0.00
Fos	-2.99	0.00
Dhh	-2.93	0.00
Pik3r3	-2.92	0.00
Mapk10	-2.67	0.00
Hal	-2.65	0.00
Lrig1	-2.37	0.00
Aldh1b1	-2.20	0.00
Cubn	-2.17	0.00
Mfge8	-2.15	0.00
Hpd	-2.15	0.00
Met	-2.13	0.00
Plk1	-2.12	0.00
Aspa	-2.08	0.00
Sspo	-1.99	0.00
Smad7	-1.98	0.00
Maob	-1.96	0.00
Maoa	-1.88	0.00
Acads	-1.81	0.00
Itgb4	-1.71	0.00
Tab1	-1.70	0.00
Aoc2	-1.64	0.00
Aacs	-1.52	0.00

Sup table 2A. Matched activated SDE TF with SDE gene in Ly6C^{high}(CT) vs Ly6C^{low}(CT) (IPA, upstream analysis, |z-score|>2, *P*<0.01)

TF	LogFC	~~State~~	z-score	p-value	Activated Targeting SDE genes
PAX5	-5.18	Inhibited	-2.81	0.00	BCL2,BLK,BLNK,CCND1,CD19,CD22,CD72,CD79A,CSF1R,CSF2RA,CTNNA1,FCER2,FLT3,FN1,G6PD,HK2,HK3,INSR,JCHAIN,MET,MMP2,PGAM1,POU2AF1,PYGL,SLC2A3,TNFRSF13C,VIM
MYC	-4.22	Inhibited	-3.54	0.00	ABCA1,ABCA7,ABCC4,ACOD1,ACSS1,ACTB,ACVR1,AHR,AKAP1,AKAP12,ALOX5,ANXA4,ANXA5,APP,AR,ATP13A2,AXL,BCL2,BCL2A1,BCR,CAMK2N1,CAPN2,CAST,Ccl6,CCNA2,CCNB2,CCND1,CCND2,CCNE1,CD151,CD19,CD274,CD44,CD69,CDC25B,CDH1,CDKN1A,CEBPA,CEBPD,CEBPE,CHRNB1,CHST15,CNP,COBLL1,COL15A1,CSF1R,CSTB,CTDSP1,CTDSPL,CTNNA1,CTSV,CXCL10,CYFIP2,CYTH2,DBI,DDB2,DDX11,DHFR,DKC1,DUSP1,DUSP2,DUSP5,DUSP6,E2F2,EBF1,ECM1,EIF2B4,EMP1,EOMES,EPCAM,EZH1,F2R,FABP4,FABP5,FAS,FASLG,Flg,FN1,FOS,G6PD,GADD45G,GATA6,GBP3,GGH,GLUD1,GLUL,GPC1,GSR,H2AZ1,HIVEP2,HK2,HLA-A, HMOX1,HMOX2,HSD11B2,HSP90AA1,ID1,IDH1,IER3,IFI16,IFI44,IFIH1,IFIT2,IFIT3,Igha,IL5RA,IQGAP2,IRF7,ITGA1,ITGAM,ITGAX,ITGB5,ITM2B,JAG2,JUN,KLF10,LAMP1,LAMP2,LDHB,LGR5,LXN,Ly6a,LYZ,MAPK3,MBP,MCM5,MCM6,MCM7,MITF,MTHFD1,MTHFR,MYBBP1A,MYC,MYD88,NCKAP1,NDRG1,NFATC3,NIBAN1,NME2,NOP58,NRP1,OAS1,OASL,Oasl2,OAT,PAX5,Pdlim3,PFKFB1,PFKM,PGAM1,PIAS4,PKM,PLAUR,PLSCR1,PML,POLD1,POLDIP3,POLR2D,POU4F1,PPAT,PPL,PRDX2,PRKCA,Prl2c2,RARA,RARG,RBBP7,RHOB,SAT1,SCARB1,SCEL,SDCBP,SEMA4A,SFXN1,SLC16A1,SLC22A4,SLC25A5,SLC2A3,SMS,SOX5,SQOR,SRM,STAT1,STMN1,SUCLA2,TAT,TCF3,TERT,TES,TESPA1,TF,TFRC,TGFB1,Tgtp1/Tgtp2,TIAM1,TIMM10B,TKT,TLN1,TLR7,TNFRSF19,TNFRSF8,TNS3,TPD52,TSPO,TWSG1,TYMS,UGT1A6,USP18,VAMP3,VARS,VIM,VPS72,WNT5A,ZFP36L1
IKZF3	-4.21	Inhibited	-2.75	0.00	ALOX5,BCL2,BLNK,CAV2,CD38,CD68,CITED2,DOCK4,FN1,IFIT3,IRF7,MAN1C1,MYC,PROS1,RASSF4,RNF213,RTP4,TNS3
TBX21	-3.40	Inhibited	-2.82	0.00	CCL3L3,CCL4,CD38,CX3CR1,CXCR3,CXCR5,EOMES,FAM89B,FASLG,GZMA,Gzmb,HLX,IFNG,IL12RB1,IL12RB2,IL18RAP,IL2RB,IL6R,KLRD1,KLRG1,Klrk1,NKG7,PRF1,STAT4,TBX21,ZEB2
EOMES	-3.33	Inhibited	-2.74	~~0.10~~	ACTA2,ANXA1,APLNR,DNAJC15,EOMES,FASLG,FREM1,GZMA,IFNG,IL2RB,ITGA2,Klra7,NRP2,PRF1,RGS2,RND3,TDRD7,TGFBI
SP110	-1.65	Inhibited	-2.30	0.00	ANXA5,ATF3,CD74,CXCL10,DAB2IP,DMD,DUSP10,EEF2K,EGR3,ETS1,F2R,Gm2016,GRN,IFIH1,IFIT3,IFITM3,IL6R,MRTFB,MTHFR,MYC,NFIL3,OAS1,OAS3,P2RX7,PLSCR1,POLE,PPARG,PRKCA,PTAFR,RAC1,RPA1,RXRA,STAT1,STAT5A,TCF3,TGFBR3,TNFRSF1A,TNFRSF21
TRIM24	-1.03	Inhibited	-4.86	0.00	AGRN,BLNK,Bst2,CA2,CALHM6,CMPK2,CXCL10,DHX58,EMB,EPSTI1,GBP3,GLUL,HERC6,Ifi27l2a/Ifi27l2b,IFI44,Ifi47,IFIH1,IFIT1B,IFIT2,IFIT3,IFNGR1,IFNGR2,Igtp,IRF7,IRGM,Irgm1,ISG15,LGALS3,LGALS3BP,Ly6a,Ms4a4b,OAS1,OASL,PARP12,PCLAF,PHF11,PLAC8,PRKCQ,PRPS2,RTP4,SAMHD1,STAT1,STAT2,TAP1,TGFBI,Tgtp1/Tgtp2,TLR2,TRAFD1,TRIM6-TRIM34,USP18
IRF5	1.09	Activated	2.93	0.00	BAK1,CCL4,CCL5,CDKN1A,CMPK2,CXCL10,DHX58,IFI44,IFIH1,IFIT2,IFIT3,IFITM3,IRF5,IRF7,ISG15,MYC,NAMPT,OAS1,OAS2,OASL,Oasl2,PARP12,PLSCR1,PRKRA,RAC1,SP110,STAT1,STAT2,TGFB1,TMPO,USP8
STAT1	1.27	Activated	3.01	0.00	ABCA1,ACOD1,ALPK1,APOBEC3B,APOL6,AXL,BAD,BATF2,BTNL9,C1R,C3,CALHM6,Ccl2,CCL3L3,CCL4,CCL5,CCND1,CCND2,CCNE1,CCR6,CCR7,CD14,CD274,CDKN1A,CEBPD,CFB,Chil3/Chil4,CLIC5,CMPK2,CSF3R,CTSS,CX3CR1,CXCL10,CXCR3,EIF2AK2,EPSTI1,FAS,FASLG,FCER1G,FCGR1A,FOS,FURIN,GBP3,GBP6,Gzmb,HERC6,HLA-DQA1,HLA-DRB5,IFI16,IFI30,IFI44,Ifi47,IFIH1,IFIT1B,IFIT2,IFIT3,IFITM2,IFITM3,IFNG,Igtp,IL12RB2,IL15,IRF5,IRF7,Irgm1,ISG15,ITGAX,JUN,Klrk1,Ly6a,MAFA,Mx1,Mx2,MYC,NFE2,OAS1,OAS2,OAS3,OASL,Oasl2,PDCD1LG2,PLSCR1,PPARG,PRF1,RNF213,RTP4,SAMHD1,Serpina3g,SLAMF8,SLC8A1,Slfn1,SLFN12L,SLFN13,Slfn2,SLFN5,SMAGP,SOAT1,SORT1,SP110,STAT1,STAT2,TAP1,TBX21,Tgtp1/Tgtp2,TLR8,TRAFD1,USP18,Wfdc17,XAF1,ZBP1
SPI1	1.41	Activated	3.74	0.00	Acp5,ACTA2,ACTB,BCL11A,BCL7A,BLNK,CCL4,CCNA2,CCNB2,CCND1,CCND2,CCR6,CCR7,CD14,CD19,CD68,CD72,CD79A,CD79B,CDKN1A,CEBPA,CEBPE,CHIT1,CMPK2,CSF1R,CSF2RA,CSF2RB,CSF3R,CTSS,CXCL10,CYBB,DUSP6,E2F2,EBF1,ETS2,FCER1G,FES,FOS,Foxp1,FUT7,GATA1,HDAC7,HK3,HSPA8,IFI44,IFIT1B,IFIT2,IFIT3,IFITM3,Iglv2,IL1RN,IL2RA,IMP4,IRF7,ISG15,ITGA5,ITGAM,JUN,Klra7,LILRB3,LMO2,LYZ,MBP,MMP2,MS4A1,MYB,MYC,NCF2,OASL,OPRM1,P2RY10,PARP12,PML,PRTN3,PTPN6,SP110,SPI1,TFEC,TLR2,TREM1,USP18,VIM
KLF10	1.48	Activated	2.21	0.04	BCL2,CCND1,CDKN1A,TBX21,TGFB1
TRPS1	1.52	Activated	2.73	~~0.07~~	ACSL1,CCNA2,CCNB2,ENPP1,FN1,GPR141,KIF11,MMP2,PACSIN1,RAMP1,SPC25
STAT2	1.80	Activated	2.69	0.00	BCL2,Ccl2,CCL5,CXCL10,GBP6,Ifi47,IFIT1B,IFIT2,IFIT3,IFITM2,IFNG,IRF5,IRF7,ISG15,Mx1,OAS1,OAS2,Oasl2,RTP4,STAT1,USP18,ZBP1
CEBPE	2.70	Activated	2.52	0.00	ALOX5AP,BCL2,Ccl9,CCND2,CCNE1,CD14,CLEC10A,CSF1R,CSF3R,CTSV,GATA1,IL1RN,IL5RA,ITGAM,LYZ,MBP,MMP2,MMP8,MYC,Ngp,RNASE2
CEBPD	2.71	Activated	2.63	0.00	ALOX5AP,BCL2A1,C3,Ccl2,CCL3L3,CCNB2,CCND1,CD14,CDKN1A,CEBPD,CSF1R,CSF3R,CTSV,FABP4,FOS,HP,ITGAM,ITGAX,MBP,MITF,MMP8,MYC,PPARG,PRTN3,PTAFR,TLR8,XBP1
IRF7	2.86	Activated	5.79	0.00	BC147527,CALHM6,Ccl2,CCL5,CD69,CMPK2,CTLA4,CXCL10,DAXX,DHX58,FCGR1A,GBP3,IFI16,IFI44,Ifi47,IFIH1,IFIT1B,IFIT2,IFIT3,IFITM2,IFITM3,Igtp,IL15,IL27,IL4I1,IRF5,IRF7,IRGM,Irgm1,ISG15,ITGAM,ITGAX,LILRA5,Ly6a,Ms4a4b,Mx1,Mx2,NAMPT,OAS1,OAS2,OAS3,OASL,Oasl2,PARP12,PARP14,PELI1,PHF11,PLAC8,PLSCR1,RTP4,SAP30,Slfn1,STAT1,STAT2,TAP1,TDRD7,TLR8,TMPO,TRAF1,TREX1,Trim30a/Trim30d,USP18,USP8,XAF1,ZBP1
CEBPA	3.10	Activated	3.14	0.00	Abcb1b,ACOD1,ACSL1,AKAP12,ALOX5AP,ANXA1,APLNR,ARL4C,BCL2,BCL2A1,C3,CA2,CCL4,Ccl9,CCNA2,CCNB2,CCND1,CCND2,CD14,CD19,CD7,CDH1,CDKN1A,CEBPA,CEBPD,CEBPE,CSF1R,CSF2RA,CSF3R,CTNNA1,DGAT2,DHFR,EBF1,EPHX1,EXTL2,F8,FABP4,FCGR1A,Flg,FLT1,FOS,G0S2,GATA6,GGH,GLIPR1,GLRX,HMOX1,HP,HSD11B1,ID1,IER3,IKZF3,IL1RN,IL6R,INSR,ISG15,ITGAM,ITGAX,ITGB5,JUN,JUNB,KLRC1,LCK,LPL,Ly6a,LYL1,MALT1,MAPKAPK2,MMP8,MSI2,MYC,NFIL3,NRP1,OAS2,OXTR,PAX5,PGD,PLIN2,PLOD2,PLXND1,PPARG,PPL,PRTN3,PTAFR,RAB31,RGS2,S100A9,SCD,SERPINI1,SMPDL3A,SPI1,SPINT2,TBXAS1,TIAM1,TNFRSF19,TNFRSF1A,VCAN,VCL,ZNF296
IFI16	4.65	Activated	2.10	0.00	BAD,CCL4,CCL5,CCND1,CD81,CDKN1A,CSF3R,CXCL10,DHFR,E2F2,IFI16,IL1RN,IL2RB,ISG15,LDLR,LPL,MGST1,Mx1,OAS1,STAT2

Sup table 2B. **SDE transcription regulator match with SDE gene in $Ly6C^{high}$ vs $Ly6C^{low}$ in *ApoE*$^{-/-}$ mice**

TFs	LogFC	z-score	p-value	Target Molecules in Dataset
Eomes	-3.50	-2.98	0.03	ACTA2,ADM,ANXA1,APLNR,CAP2,EOMES,FASLG,FAT3,GZMA,IFNG,IL2RB,ITGA2,Klra7,NRP2,POU5F1,PRF1,RND3,TBX6,TDRD7,TGFBI,TNFSF10,ZNF521
Myc	-3.47	-2.57	0.00	ABCA7,ABCC3,ABCC4,ACOD1,ACSS1,ACTN1,ACVR1,ADK,ADM,AFP,AHR,ALDH18A1,ANXA4,ANXA5,APP,AR,ARG1,ASCL2,AURKB,AXL,BCKDHB,BCL2,BCL2A1,BCR,BMPR1A,BRCA1,C12orf49,CAMK2N1,CAPN2,Ccl6,CCNB1,CCNB2,CCND1,CCND2,CCNE1,CD19,CD274,CDC25B,CDCA7,CDH1,CDKN1A,CEBPA,CEBPD,CHEK1,CHRNB1,CHST15,CIAO2A,CNP,COBLL1,COL14A1,COL4A2,COMMD3BMI1,CPT1A,Crisp1/Crisp3,CRYAB,CSTB,CTDSPL,CTSV,CXCL10,CYFIP2 DBI,DDX11,DKC1,DUSP2,DUSP5,DUSP6,DUSP7,E2F1,E2F2,Ear2,EBF1,EBI3,EGR1,EGR2,EIF4EBP1,EMP1,ENO1,EOMES,EPCAM,F2R,FABP4,FABP5,FASLG,Flg,FN1,FOS,FOXM1,FSTL1,G6PD,GADD45G,GAMT,Gar1,GATA6,GBP3,GDF3,GGH,GLUD1,GLUL,GPC1,GSR,H2AZ1,HES1,HIVEP2,HK2,HLAA,HMOX2,HSPB1,ID1,IDH1,IDH2,IER3,IFI16,IFI44,IFIH1,IFIT1,IFIT2,IFIT3,Igha,IL10,IL17RB,IL5RA,IMPA2,IMPACT,IQGAP2,IRF7,ITGA1,ITGA3,ITGAX,JUN,KHDC3L,LAMP1,LAMP2,LDHB,Ly6a,LYZ,MAPK3,MCM6,MCM7,MITF,MRC1,MSH2,MTBP,MTHFD1,MTHFR,MYC,MYD88,MYLPF,NCAM1,NDRG1,NFAT5,NFATC3,NIBAN1,NME2,NRP1,OAS1,OASL,Oasl2,PAK1,PAX5,PCDH18,Pdlim3,PECAM1,PFKFB1,PGK1,PIAS3,PLAU,PLP1,PLSCR1,POLD1,POU5F1,PPAT,PPL,PRDX4,PRKCA,PYCR1,RARA,RARG,RBBP7,RHOB,RRM2,SARDH,SCARB1,SCEL,SEMA4A,SHMT2,SLC16A1,SLC22A4,SLC25A5,SMN1/SMN2,SMS,SOX5,SPN,SPP1,SQOR,SRM,STMN1,TAT,TCF3,Tcf7,TERT,TES,TESPA1,TF,TFRC,TGM1,THOP1,TIAM1,TIMM10B,TNFRSF19,TNFRSF8,TNFSF10,TNS3,TPD52,TSPO,TWSG1,UGT1A1,UGT1A3,UGT1A6,USP18,VEGFC,VIM,ZFP36L1
Pax5	-3.45	-3.06	0.00	BCL2,BLK,BLNK,CCND1,CD19,CD22,CD72,CD79A,FCER2,FN1,G6PD,HK2,JCHAIN,MET,POU2AF1,PTPRF,PYGL,SDC1,TNFRSF13C,VIM
Tbx21	-2.96	-2.53	0.00	CCL3L3,CCL4,CD38,CXCR3,CXCR5,EOMES,FAM89B,FASLG,GATA3,GZMA,Gzmb,HLX,ICOS,IFNG,IL12RB1,IL12RB2,IL18RAP,IL2RB,IL6R,IL7R,ITGAE,ITK,KLRD1,KLRG1,Klrk1,NKG7,PRF1,PTGDR2,SELL,SPP1,STAT4,TBX21,Tcf7
Mybl2	1.57	-2.17	0.00	BCL2,CCNB1,CCNB2,CCND1,CDC25B,CEBPA,CLTA,FASLG,FOXM1,GATA2,ID1,IFNG,IGFBP5,MYBL2,MYC,TFEC,TOP2A
Pou5f1	1.62	-2.18	0.00	ACVR1,AFP,AHR,B3GNT7,BCL2,BCL2A1,BMPR1A,BNIP3L,BRCA1,CARD6,CCND1,CDH1,CDKN1A,CHST15,COBLL1,CXCL10,DAPK1,DFFA,DUSP5,EOMES,EPCAM,FASLG,Flg,FUT4,GADD45G,GATA2,GATA6,GBP3,GDF3,HOPX,IER5L,IL6,KHDC3L,LOXL1,LTA,MYBL2,MYC,NCAM1,NRP1,Oasl2,PCDH18,PCGF6,Pdlim3,PECAM1,PHC1,PMEPA1,POLE4,POU5F1,PPL,RARA,SIRPA,SPP1,TDRD7,TF,TGM1,TNFRSF19,TNFRSF1A,TNFRSF21,TNFRSF9,TNFSF10,TNFSF8,TRAF4,TWSG1,USP18,VIM,ZEB1
Prdm16	1.80	-2.33	0.00	CCL5,CDKN1A,CXCL10,Cxcl9,GADD45G,GBP3,GPX3,HGF,IFI44,IFIT2,IRF7,Mx2,OAS2,OAS3,PPARGC1A
Tcf7	-1.83	2.60	0.00	BCL2,CCL3L3,CCL4,DTX1,EOMES,FASLG,FYN,GATA3,GZMA,IFNG,IL2RA,IL7R,Klra7,LEF1,SOX12,TBX21,TNFRSF19,TOX
Irf5	1.02	2.98	0.00	CCL4,CCL5,CDKN1A,CMPK2,CXCL10,DAPK2,DHX58,FOXO1,IFI44,IFIH1,IFIT1,IFIT2,IFIT3,IFITM3,IL12A,IL6,IRF5,IRF7,ISG15,MYC,NAMPT,OAS1,OAS2,OASL,Oasl2,PARP12,PLSCR1,PRKRA,SP110,TMPO,TNFSF10
Trps1	1.06	3.01	0.00	ALPL,AURKB,CCNB2,CENPF,ENPP1,ENPP6,FN1,FOXM1,GALNT3,GPR141,KIF11,PACSIN1,RAMP1,SERPINB2,TOP2A,ZEB1
Cebpd	2.06	2.28	0.00	ALOX5AP,BCL2A1,BRCA1,C3,Ccl2,CCL3L3,CCNB1,CCNB2,CCND1,CDKN1A,CEBPD,CSF3R,CTSV,FABP4,FOS,HGF,HP,IGFBP5,IL10,IL6,ITGAX,MITF,MMP8,MYC,PPARG,PRTN3,PTAFR,TLR8,VEGFC,XBP1
Irf7	2.16	4.87	0.00	LJJL;Q16IKLN NJ MHJ
Cebpa	2.67	2.55	0.00	ABCB1,Abcb1b,ACOD1,AKR1C3,ALOX5AP,ANPEP,ANXA1,APLNR,ARG1,ARG2,ARL4C,ASL,BCL2,BCL2A1,C3,C3AR1,CCL4,Ccl9,CCNB2,CCND1,CCND2,CD19,CDH1,CDKN1A,CEBPA,CEBPD,CPT1A,CSF3R,CTSK,CYP11A1,DGAT2,E2F1,EBF1,EFNB2,EGR2,EPHX1,EXTL2,FABP4,FCGR1A,Flg,FLT1,FOS,FOXM1,FOXO1,FXR2,G0S2,GATA2,GATA6,GGH,GLRX,HGF,HP,HSD11B1,ICAM2,ID1,IER3,IKZF3,IL10,IL1RN,IL6,IL6R,ISG15,ITGAX,JUN,KLRC1,LCK,LCN2,LPL,Ly6a,MAPKAPK2,MMP8,MSI2,MYBL2,MYC,NFIL3,NR4A2,NRP1,OAS2,OLR1,Orm1,PAX5,PGD,PLIN2,PLOD2,PLXND1,PPARG,PPARGC1A,PPL,PRTN3,PTAFR,RAB31,RARRES2,RIN1,S100A8,S100A9,SCD,SERPINB2,SERPINF1,SERPINI1,SMPDL3A,SPINT2,SPP1,STAR,TBXAS1,THRA,TIAM1,TNFRSF19,TNFRSF1A,TNFSF10,TRAF4,VCAN,VCL,ZBTB48,ZNF296

Supplementary table 3

Sup. table 3. Reported MC generation TF (literature screening)

TFs	Gene Symbol	Gene full name	PMID	Gene ID	
				Hs	Mm
IRF8	Irf8	interferon regulatory factor 8	23319570	3394	15900
PU.1	Spi1	spleen focus forming virus (SFFV) proviral integration oncogene	9687512	6688	20375
FLI1	Fli1	Friend leukemia integration 1	15367440	2313	30619
KLF4	Klf4	Kruppel like factor 4	18390749	9314	16600
KLF2	Klf2	Kruppel like factor 2	28379391	10365	16598
NR4A1	Nr4a1	nuclear receptor subfamily 4 group A member 1	21725321	3164	15370
C/EBPβ	Cebpb	CCAAT/enhancer binding protein (C/EBP)	28514690	1051	12608
TET3	Tet3	Ten-Eleven-Translocation-3	30575719	00424	194388
c-Jun	Jun	jun proto-oncogene	23525665	3725	16476
JunB	Junb	jun B proto-oncogene	23525665	3726	16477
STAT1	Stat1	signal transducer and activator of transcription 1	23525665	6772	20846
STAT3	Stat3	signal transducer and activator of transcription 3	23525665	6774	20848
VDR	Vdr	vitamin D receptor	23525665	7421	22337
IRF1	Irf1	interferon regulatory factor 1	23525665	3659	16362
IRF4	Irf4	interferon regulatory factor 4	23525665	3662	16364
C/EBPε	Cebpe	CCAAT/enhancer binding protein (C/EBP), epsilon	23525665	1053	110794
C/EBPα-p42	Cebpa	CCAAT/enhancer binding protein (C/EBP), alpha	9012825	1050	12606
GATA-2	Gata2	GATA binding protein 2	25707267	2624	14461
RUNX1	Runx1	runt related transcription factor 1	17994017	861	12394
TCF19	Tcf19	transcription factor 19	27386937	6941	106795
POU5F1	Pou5f1	POU domain, class 5, transcription factor 1	27386937	5460	18999
